# Role of NLRP3 Inflammasome in Heart Failure Patients Undergoing Cardiac Surgery as a Potential Determinant of Postoperative Atrial Fibrillation and Remodeling: Is SGLT2 Cotransporter Inhibition an Alternative for Cardioprotection?

**DOI:** 10.3390/antiox13111388

**Published:** 2024-11-14

**Authors:** Rodrigo L. Castillo, Jorge Farías, Cristian Sandoval, Alejandro González-Candia, Esteban Figueroa, Mauricio Quezada, Gonzalo Cruz, Paola Llanos, Gonzalo Jorquera, Sawa Kostin, Rodrigo Carrasco

**Affiliations:** 1 Departamento de Medicina Interna Oriente, Facultad de Medicina, Universidad de Chile, Santiago 7500922, Chile; 2 Unidad de Paciente Crítico, Hospital del Salvador, Santiago 7500922, Chile; 3 Departamento de Ingeniería Química, Facultad de Ingeniería y Ciencias, Universidad de La Frontera, Temuco 4811230, Chile; 4 Escuela de Tecnología Médica, Facultad de Salud, Universidad Santo Tomás, Los Carreras 753, Osorno 5310431, Chile; cristian.sandoval@ufrontera.cl; 5 Departamento de Medicina Interna, Facultad de Medicina, Universidad de La Frontera, Temuco 4811230, Chile; 6 Instituto de Ciencias de la Salud, Universidad de O’Higgins, Rancagua 2841959, Chile; alejandro.gonzalez@uoh.cl (A.G.-C.); esteban.figueroa@uoh.cl (E.F.); 7 Facultad de Medicina, Universidad Finis Terrae, Santiago 7501015, Chile; mauricioquezadac@gmail.com; 8 Instituto de Fisiología, Facultad de Ciencias, Universidad de Valparaíso, Valparaíso 2360102, Chile; gonzalo.cruznec@gmail.com; 9 Centro de Estudios en Ejercicio, Metabolismo y Cáncer, Facultad de Medicina, Universidad de Chile, Santiago 8380453, Chile; pllanos@odontologia.uchile.cl; 10 Facultad de Odontología, Instituto de Investigación en Ciencias Odontológicas, Universidad de Chile, Santiago 8380544, Chile; 11 Instituto de Nutrición y Tecnología de los Alimentos (INTA), Universidad de Chile, Santiago 8331051, Chile; gonzalo.jorquera@inta.uchile.cl; 12 Faculty of Health Sciences, Brandenburg Medical School Theodor Fontane, 16816 Neuruppin, Germany; costin.sava@mhb-fontane.de; 13 Departamento de Cardiología, Clínica Alemana de Santiago, Santiago 7500922, Chile; rcarrascoloza@gmail.com

**Keywords:** postoperative atrial fibrillation, heart failure with preserved ejection fraction, oxidative stress, NLRP3 inflammasome, ventricular remodeling, SGLT2 inhibitors

## Abstract

In heart failure (HF) patients undergoing cardiac surgery, an increased activity of mechanisms related to cardiac remodeling may determine a higher risk of postoperative atrial fibrillation (POAF). Given that atrial fibrillation (AF) has a negative impact on the course and management of HF, including the need for anticoagulation therapy, identifying the factors associated with AF occurrence after cardiac surgery is crucial for the prognosis of these patients. POAF is thought to occur when various clinical and biochemical triggers act on susceptible cardiac tissue (first hit), with oxidative stress and inflammation during cardiopulmonary bypass (CPB) surgery being potential contributing factors (second hit). However, the molecular mechanisms involved in these processes remain poorly characterized. Recent research has shown that patients who later develop POAF often have pre-existing abnormalities in calcium handling and activation of NLRP3-inflammasome signaling in their atrial cardiomyocytes. These molecular changes may make cardiomyocytes more susceptible to spontaneous Ca2+-releases and subsequent arrhythmias, particularly when exposed to inflammatory mediators. Additionally, some clinical studies have linked POAF with elevated preoperative inflammatory markers, but there is a need for further research in order to better understand the impact of CPB surgery on local and systemic inflammation. This knowledge would make it possible to determine whether patients susceptible to POAF have pre-existing inflammatory conditions or cellular electrophysiological factors that make them more prone to developing AF and cardiac remodeling. In this context, the NLRP3 inflammasome, expressed in cardiomyocytes and cardiac fibroblasts, has been identified as playing a key role in the development of HF and AF, making patients with pre-existing HF with reduced ejection fraction (HFrEF) the focus of several clinical studies with interventions that act at this level. On the other hand, HFpEF has been linked to metabolic and non-ischemic risk factors, but more research is needed to better characterize the myocardial remodeling events associated with HFpEF. Therefore, since ventricular remodeling may differ between HFrEF and HFpEF, it is necessary to perform studies in both groups of patients due to their pathophysiological variations. Clinical evidence has shown that pharmacological therapies that are effective for HFrEF may not provide the same anti-remodeling benefits in HFpEF patients, particularly compared to traditional adrenergic and renin–angiotensin–aldosterone system inhibitors. On the other hand, there is growing interest in medications with pleiotropic or antioxidant/anti-inflammatory effects, such as sodium–glucose cotransporter 2 inhibitors (SGLT-2is). These drugs may offer anti-remodeling effects in both HFrEF and HFpEF by inhibiting pro-inflammatory, pro-oxidant, and NLRP3 signaling pathways and their mediators. The anti-inflammatory, antioxidant, and anti-remodeling effects of SGLT-2 i have progressively expanded from HFrEF and HFpEF to other forms of cardiac remodeling. However, these advances in research have not yet encompassed POAF despite its associations with inflammation, oxidative stress, and remodeling. Currently, the direct or indirect effects of NLRP3-dependent pathway inhibition on the occurrence of POAF have not been clinically assessed. However, given that NLRP3 pathway inhibition may also indirectly affect other pathways, such as inhibition of NF-kappaB or inhibition of matrix synthesis, which are strongly linked to POAF and cardiac remodeling, it is reasonable to hypothesize that this type of intervention could play a role in preventing these events.

## 1. Introduction

The proportion of patients suffering postoperative atrial fibrillation (POAF) may be as high as 64% following procedures for valvular pathology [1]. POAF is a significant complication that may lead to hemodynamic instability, thromboembolism, transient ischemic attack, stroke, end-organ failure, prolonged hospital stays, increased mortality, and increased healthcare costs [2]. It is associated with increased postoperative mortality and a significant decrease in long-term survival rates [3]. Potential predisposing factors that have been implicated include age, type of cardiac surgery, atrial distension, and pre-existing cardiac conditions [4]. Others perioperative factors include mechanical deformation, which can cause sympathetic activation, and hemodynamic and hypoxic injuries involved with intraoperative extracorporeal circulation, such as anemia, prolonged reperfusion time, and insufficient analgesic support. On the other hand, hypervolemia induction may also determine atrial fibrillation susceptibility by increasing the atrial volume and structural remodeling [5]. Myocardial injury due to surgery and pericardial inflammation or early cytokines releases (i.e., IL-6) have also been implicated as potential pathogenetic AF mechanisms [6]. However, since no single pathogenetic mechanism seems to be solely responsible for developing OAF, a comprehensive set of biochemical and hematologic parameters must be assessed. Low left ventricular (LV) function has been used as a predictor marker of worse postoperative evolution, mortality, and cardiovascular outcome in patients who develop POAF [7,8]. Echocardiographic measures of LV function, which have been used in cardiology hospitalized patients, have failed to penetrate the perioperative setting because of practicality, and calculations require concomitant findings (e.g., mitral regurgitation for dP/dt). Moreover, using hemodynamic monitoring data, it is possible to calculate inotropy and kinetic energy values after a cardiopulmonary bypass and to understand associations with postoperative outcomes [9]. Recently, LV systolic dysfunction by global longitudinal strain (GLS), a predictor of cardiovascular events, may predict new-onset AF in a population setting [10,11]. Global longitudinal strain assessment may improve AF risk stratification and other established parameters, such as total atrial conduction and increased left atrial volume indexed for body surface area [12]. Based on the speckle tracking method and the direct determination of the myocardial deformation, the myocardial strain could reflect myocardial contractility. This variable strongly assesses the contractile function and determines the association with HF outcome occurrence independent of left ventricular function. For example, AF induction can be predicted by subclinical atrial deformation or strain changes [13]. In this context, the ventricular strain is a sensitive measurement for detecting subclinical impairment, and it may be used to predict the development of POAF. Also, a recent trial conducted to test mortality and hospitalizations in patients with the diverse cardiac disease found that ventricular GLS independently predicted the mortality compared with LVEF in near 6000 patients with HFrEF, acute myocardial infarction, and valvular pathologies [14]. In patients with HFpEF, GLS is a potential predictor of heart failure-related hospitalizations, cardiovascular death, and other clinical outcomes [15,16]. Examining the POAF predictors in patients with preoperative subclinical ventricular dysfunction and the complex pathophysiology of acute HFpEF coupled with inadequate stratification tools and a lack of available therapies provides the rationale for assessing the utility of LV GLS in these clinical settings [17]. Also, oxidative stress and pro-inflammatory biochemical factors that determine contractile impairment are ongoing in study. However, the recent basic and clinical studies are not clear in regard to estimating the strong relationship with the preoperative cardiovascular function and POAF occurrence; therefore, only it must be presented as a theorical hypothesis. In this case, we attempt, in this review, to provide and analyze data on the effect of a promising pharmacological intervention with SGLT-2is in other medium- and long-term cardiovascular settings, such as cardiac surgery with a CPB.

## 2. Oxidative Stress and Inflammation as a Mediator of POAF

In 2001, the existence of oxidative alterations in samples of atrial tissue from patients with chronic atrial fibrillation (AF) was demonstrated: an increase in oxidative modifications in various proteins present in cardiomyocytes was evidenced compared to samples from patients without AF [18]. The same research group demonstrated in a subsequent study involving an animal model that induced atrial tachycardia (atrial tachi-pacing) decreased the concentration of ascorbic acid in atrial tissue while simultaneously causing an increase in the content of nitrated proteins, with both situations reflecting a state of oxidative stress (OS) [19]. Other researchers have shown that the presence of superoxide anion from NOX-2 has been associated with the onset of AF [20] but not its maintenance [21]. In 2023, Hansen et al., sought to investigate the effect of OE at the onset of reperfusion and its involvement in developing IR-associated arrhythmias in a porcine model of myocardial infarction. They found that levels of malondialdehyde (MDA), a marker of lipid peroxidation, and 3-nitrotyrosine (3NT), a marker of protein oxidation, are increased at the tissue level as early as 5 min into reperfusion compared to baseline control [22]. Moreover, the increase in these markers was not attenuated by use of antioxidant agents such as N-acetylcysteine. In the same study, the total number of arrhythmias showed no significant differences between the control and N-acetylcysteine groups.

Different pathophysiological events associated with cardiac surgery imply an injury against the cardiac tissue, triggering the formation of a local ROS burst. Several mechanisms, including enzymatic and non-enzymatic inductions such as mitochondrial dysfunction, nicotinamide adenine dinucleotide phosphate (NADPH)-oxidase activation, Fenton reaction, Tetrahydrobiopterin depletion, and lower GTP cyclohydrolase 1 activity (enzyme activity can lead to higher BH4 levels and increased nitric oxide production), generate an ROS burst in the early phase of reperfusion [23,24,25]. This biochemical event, associated with decreased heart antioxidant mechanisms, makes the myocardial tissue extremely vulnerable to oxidative damage and pathophysiological events [26,27]. In patients undergoing cardiac surgery with a cardiopulmonary bypass (CPB), pro-oxidant tissue damage has been associated with a functional or structural pathophysiological substrate for developing POAF [28,29]. For example, ROS-production-derived NADPH-oxidase activation in atrial tissue is an independent predictor of postoperative AF, suggesting that this local oxidase system determined a pro-oxidant imbalance upon atrial oxidative stress [30,31]. In the same way, biomarkers of oxidative stress concentration in urine, isoprostane, and isofluorane showed a relatively linear correlation with POAF in cardiac surgery patients [32]. In addition, preclinical data indicate that this pro-oxidant imbalance during reperfusion time contributes to progressive cardiac disfunction. Initially, the local inflammation determines a recruitment of polymorphonuclear (PMN) neutrophils and higher ROS concentration, triggering matrix infiltration and initial structural remodeling. Cellular events included cardiomyocyte death and coronary microvascular disturbances, which enhance the consequences of pathological remodeling [28,33,34]. Both left ventricular cellular and structural dysfunction is strongly linked to sterile inflammation and immune cell recruitment in damaged cardiac tissue. These cellular remodeling events are demonstrated in acute myocardial infarction and secondary reperfusion processes in the ischemic HFrEF [35]. However, this type of remodeling and its biochemical markers associated with the medium or long-term outcomes in patients with HFpEF are not yet well characterized. Many previous reports have shown that inflammatory cytokines contribute to the pathogenic process of arrhythmogenesis in many ways. The direct cardiac effects include prolongation of the ventricular action potential, which is a consequence of changes in the functioning of ionic channels; pathologic modifications in calcium metabolism, potentially contributing to the development of delayed afterdepolarizations; and changes in action potential conduction velocity, given by modifications in connexins which, in turn, modify the gap junction’s activity and fibrosis process, both of them being associated with heterogeneous cardiac conduction and the development of re-entry [36].

Existing clinical data associate the level and activity of white blood cells with POAF incidence. Patients who have higher PMN neutrophil counts after surgery are significantly more susceptibility to developing electrical instability and POAF [28,37,38], and patients developing POAF tend to have a greater activation of PMN and decreased electrical conduction velocity in comparison to mice treated with angiotensin II, a known arrhythmogenic stimulus [39]. Moreover, the elevated preoperative PMN neutrophils count and HATCH score in patients subjected to cardiac surgery with coronary bypass are associated with higher occurrence of POAF [31,40]. In the same way, patients undergoing cardiac surgery with preoperative [41] and perioperative [2] elevation with pro-inflammatory biomarkers such as IL-6 and CRP have a higher risk of POAF. The exact way these blood components can trigger POAF and their relationship with the cardiac inflammatory process are unknown. Previous studies using animal and cellular approaches have shown that, when neutrophils bind to ventricular myocytes, they determine arrhythmogenic mechanisms based on the induction of depolarization of the resting membrane potential and a marked prolongation of the myocyte action potential [42,43]. Consistent with these findings, the use of anti-inflammatory agents such as colchicine has been shown to reduce the pro-inflammatory and pro-fibrotic activity induced by pericarditis and to prevent POAF in these models [44]. Conversely, recent evidence indicates that novel pro-inflammatory intracellular sensors that have been previously described, such as the NLRP3 inflammasome, could determine the level of inflammatory response and their association with clinical outcomes in AF patients [45]. In addition, some genetic variant (rs10754555) of inflammasome activation is associated with increased systemic inflammation, prevalent coronary artery disease, and mortality [46,47]. In an explorative gene-centric approach, these findings were described in mainly 500,000 patients from a German cohort who were all below 60 years old, where rs10754555 obtained in blood cell carriers had a significantly higher risk for cardiovascular mortality during follow-up. In this way, in sterile pericarditis surgery rat models, intramyocardial injection of extracellular vesicles (EVs), which have known anti-inflammatory and anti-fibrotic effects, obtained from human patients has been shown to limit the pericarditis pro-fibrotic effect, reduce the infiltration with immune cells and, and in addition to this, decrease the likelihood of POAF development [48]. In another study by this group, treatment with EVs also decreased the NLRP3 inflammasome activation and the myeloperoxidase activity, showing anti-inflammatory and antioxidative effects [49]. Thus, considering the available evidence, it is reasonable to suggest a link between local and systemic inflammation during cardiac surgery due to NLRP3 inflammasome activation. From this paradigm, we can argue for the pathophysiological role of the NLRP3 inflammasome pathway in the progression to a persistent inflammatory state in terms of myocardial remodeling in patients with HFpEF.

## 3. Ischemia–Reperfusion and Cardiac Remodeling

**Figure 1:**
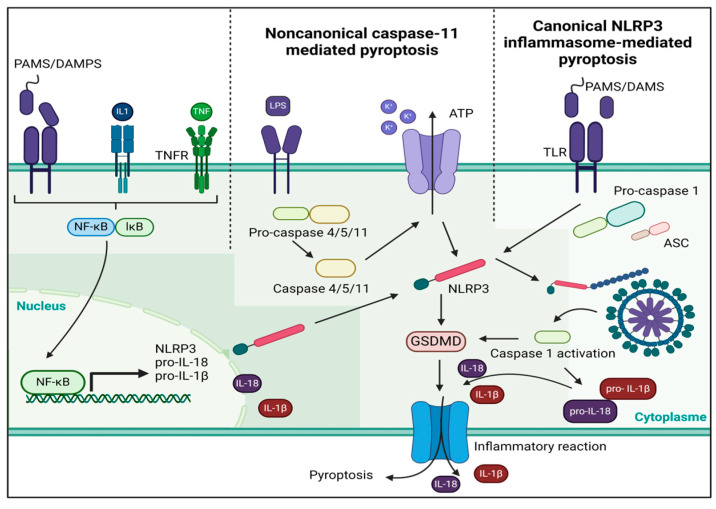
A dual-signal mechanism mediates the activation of the NLRP3 inflammasome. The first step is to help nuclear factor-κB move into the nucleus so that it can control the NLRP gene’s transcription and translation. This allows the gene to be released into the cytoplasm. In the second stage, there are two pathways: one is a non-canonical caspase-11 pathway, and the other is a canonical NLRP3 inflammasome pathway. PAMS, pathogen-associated molecular patterns, DAMPS, damage-associated molecular patterns; ATP, adenosine triphosphate; GSDMD, Gasdermin D; TLR, Toll-Like receptor.

Postoperative atrial fibrillation is a frequent arrhythmia after a CPB, and it is used as a functional and clinical outcome in patients undergoing cardiac surgery with a CPB [31,50]. However, some patients demonstrate AF persistence after the initial POAF episode, with 40% of POAF patients presenting AF recurrence. Studies indicate that AF on the 30th postoperative day affects between 2% and 5% of patients [51]. In this group of patients, it is assumed that electrical and structural remodeling processes could be relevant in determining the evolution of ventricular function and cardiovascular mortality [52]. A recent study found a higher AF recurrence rate following a rhythm control strategy in patients presenting left ventricle hypertrophy and diastolic dysfunction [53]. A worse recovery from diastolic dysfunction in HFpEF imposed by LV hypertrophy could mean a higher LA pressure and dilation, culminating in a higher arrhythmic substrate [54]. In both types of HF, it is highly relevant to investigate the molecular mechanisms by which cardiac surgery with CPB could determine pathological remodeling to establish control points with some markers and possible pharmacological therapies. The development of ventricular remodeling (VR) is a dynamic process, but the main underlying changes are found in cardiac structures, including cardiomyocytes apoptosis, cardiac fibrosis, vascular endothelial injury, and changes in extracellular matrix composition [55,56]. The NLRP3 inflammasome activation pathway employs a dual-signal model for mediation (Figure 1) [57].

During episodes of acute myocardial ischemia, there is an increase in the outflow of potassium from heart muscle cells and a decrease in the inflow of potassium into these cells. The primary mechanism of potassium outflow in cardiomyocytes during acute ischemia is an open ATP-sensitive potassium channel which aids accumulating extracellular potassium [58]. The relationship between ischemia-induced potassium efflux, sodium-activated potassium channels, and free fatty acid potassium channels has been shown [59,60]. ATP catalyzes the reduction in phosphorylation levels during acute myocardial infarction while also causing impairment in the sodium–potassium pump, decreasing the cellular potassium influx [61,62].

The initial observation of potassium promoting the activation of IL-1β was made in macrophages that were stimulated by intracellular lipopolysaccharide [63]. Subsequent research has verified that the NLRP3 inflammasome can be stimulated by acute ischemia, ATP, nigericin, and crystalline matter, resulting in the release of intracellular potassium and subsequent activation of the NLRP3 inflammasome [58,64]. The activation of the NLRP3 inflammasome is known to be influenced by changes in potassium concentration gradients in cardiomyocytes [62,65]. An elevation in intracellular anaerobic glycolysis diminishes the cytoplasmic potential of hydrogen (pH) and augments the concentration of hydron during acute myocardial infarction. The intracellular hydron is transported to the extracellular space via the sodium–hydron exchanger, increasing the intracellular sodium concentration. The sodium–calcium exchanger is responsible for maintaining equilibrium in the intracellular sodium content by removing excess sodium from the cell and bringing in external calcium. This process leads to an accumulation of calcium within the cell, resulting in a calcium overload [66]. Furthermore, apart from the transport of calcium outside the cell, the release of calcium from the internal sarcoplasmic reticulum is an additional cause of calcium overload. Sarcoplasmic reticulum serves as the primary intracellular storage site for calcium ions. Acute hypoxia causes the generation of a significant quantity of oxygen-free radicals in cardiomyocytes. These radicals harm the SR membrane, which then prompts the release of calcium into the cytoplasm and worsens calcium excess. Following reperfusion, the decreased level of extracellular hydron stimulates the exchange of sodium–hydron and sodium–calcium, exacerbating calcium overload [65,66].

## 4. NLRP3 Activity as a Mediator of Cardiovascular Damage

Inflammation is a “double-edged sword” in the body’s defense mechanism that is capable of protecting tissues and causing harm [67]. Regulated short-term inflammation may act as a protective response, whereas uncontrolled and low-grade inflammation perpetuates various disease types [68]. Low-grade systemic inflammation, characterized by chronic subtle elevations in circulating pro-inflammatory markers, has emerged as a hallmark of chronic diseases such as obesity, diabetes, and cardiovascular disorder, among others [69,70]. Overactivation of the cytosolic NLRP3 inflammasome is implicated in chronic low-grade inflammation, and it serves as a sensor of cellular stress and damage through various microbial, stress, and damage signals, leading to the direct activation of caspase-1 and triggering the subsequent release of pro-inflammatory cytokines and a type of cell death known as pyroptosis [71]. Initially, NLRP3 inflammasome was recognized due to gain-of-function mutations in the encoding gene linked with autoinflammatory cryopyrin-associated periodic syndromes [72]. The NLRP3 inflammasome consists of the sensor NLRP3 (also referred to as cryopyrin or NALP3), the adaptor apoptosis-associated speck-like protein (ASC), and the effector cysteine protease procaspase-1 [73]. NLRP3 features a C-terminal leucine-rich repeat (LRR) domain, a central NACHT domain containing ATPase that facilitates oligomerization, and an N-terminal pyrin (PYD) domain that recruits proteins necessary for inflammasome complex assembly [74,75]. ASC is a bipartite complex consisting of a PYD and a CARD which acts as a bridge that connects the sensing function of NLRP3 with the functional role of procaspase-1 [76,77].

NLRP3 activity has emerged as a critical mediator of cardiovascular damage, playing a central role in the inflammatory processes associated with various cardiovascular conditions [78]. The activation of the NLRP3 inflammasome contributes to endothelial dysfunction and hypertension [79,80], vascular inflammation [81], and atherosclerosis [82], thereby promoting the development and progression of cardiovascular diseases [83]. In the context of hypertension, it was found that a 14-day Ang II infusion caused elevated blood pressure with increased expression of NLRP3 in the aorta, as well as higher serum levels of IL-1β in mice. Then, researchers performed a similar procedure in NLRP3-/- mice and found a reduction in blood pressure elevation in response to Ang II. They also found that NLRP3 KO mice experienced less vascular oxidative stress and had improved endothelium-dependent relaxation functions [80]. The use of MCC950, a diaryl sulfonylurea-containing compound that was shown to potently and selectively inhibit the oligomerization and activation of the NLRP3 inflammasome, effectively reduced blood pressure, kidney inflammation, and fibrosis, and it also improved urine output and other markers of renal function in a murine model of salt-induced hypertension [84].

In 2010, Duewell et al., showed that animals that were genetically modified to be prone to developing atherosclerosis and who were receiving bone marrow transplants from NLRP3-deficient, ASC-deficient, or IL-1αβ/b-deficient donors were resistant to developing atheroma plaques in response to a HFD [85]. Further investigations revealed that the areas of atherosclerotic plaques in the entire aortas and aortic roots of Apoe-/-Casp1-/- mice fed a Western diet were notably diminished in comparison to those observed in Apoe-/- mice [86]. However, other reports did not find that NLRP3, ASC, or caspase 1 contributed to atherosclerosis progression, infiltration of plaques by macrophages, or plaque stability in ApoE-/- mice models [87]. The differences in gender, feeding conditions and the choice of experiment models explain these results [88]. Data collected from 555 patients who experienced myocardial infarction and 1016 healthy individuals reveals a notable elevation in the expression of NLRP3, ASC, caspase-1, IL-1β, and IL-18 mRNA within atherosclerotic plaques compared to healthy arteries. Additionally, the expression of NLRP3 mRNA was markedly higher in plaques from symptomatic patients than in those from asymptomatic individuals [89].

The induction of the inflammasome in the cardiac tissue, both during ischemic and non-ischemic injuries, represents a dysregulated response to sterile injury. This activation promotes adverse cardiac remodeling and can lead to heart failure [90]. Understanding the molecular pathophysiology behind NLRP3 activation is crucial for addressing cardiovascular issues. Moreover, recent studies have emphasized the complex relationship between NLRP3 inflammasome activation and the progression of heart failure [91]. Toldo et al. suggest that NLRP3 inflammasome activation in cardiac cells contributes to myocardial remodeling and dysfunction, representing a key pathogenic mechanism in heart failure [83]. NLRP3 KO mice resulted in a significant decrease in IL-1β production, along with reduced cardiac hypertrophy and impaired contractile function under pressure overload conditions. Pharmacological depletion of extracellular ATP or genetic disruption of the P2X7 receptor suppressed myocardial NLRP3 inflammasome activity during pressure overload, underscoring the pivotal role of the NLRP3/ATP/P2X7 axis in cardiac inflammation and hypertrophy. Notably, extracellular ATP induced hypertrophic changes in cardiac cells in vitro in an NLRP3- and IL-1β-dependent manner [92]. In a clinical study, thirty-one patients diagnosed with heart failure with preserved ejection fraction and CRP (C-reactive protein) levels > 2 mg/L were randomly assigned to receive either anakinra (a recombinant IL-1 receptor antagonist) at a daily subcutaneous dose of 100 mg (N = 21) or a placebo (N = 10) for a duration of 12 weeks. Despite the administration of anakinra over this period, there was no observed improvement in peak oxygen consumption (VO_2_) or ventilatory efficiency among obese patients with heart failure and preserved ejection fraction. However, among those patients treated with anakinra, there was a notable decrease in levels of high-sensitivity C-reactive protein and N-terminal proBNP after 4 weeks compared to baseline. The reduction in NT-proBNP levels due to treatment is generally considered as a positive prognostic sign. The encouraging trends observed in high-sensitivity CRP and NT-proBNP levels with anakinra warrant further investigation in subsequent studies [93].

These results emphasize the potential therapeutic value of targeting NLRP3 signaling pathways in mitigating cardiovascular damage and improving heart failure outcomes. Furthermore, dysregulation of NLRP3 activity has been implicated in the pathogenesis of acute myocardial infarction (AMI). Activation of the NLRP3 inflammasome exacerbates myocardial injury following AMI, promoting adverse cardiac remodeling and increasing the risk of subsequent cardiovascular events [78,90]. The formation of inflammasomes during ischemia/reperfusion (I/R) and their subsequent activation have been reported, leading to the production of interleukin-1β and triggering inflammatory processes such as infiltration of inflammatory cells and the expression of cytokines in the heart. In mice deficient in ASC and caspase-1, these inflammatory responses and consequent injuries, including infarct formation, myocardial fibrosis, and dysfunction, were significantly reduced [94]. The use of the covalent NLRP3 inhibitor oridonin preserved left ventricular ejection fractions and fractional shortening, and it also markedly limited the myocardial infarct size in surgically induced myocardial infarction in mice [95]. The protein levels of NLRP3, IL-1β, and IL-18 were reduced in the myocardium and blood of the oridonin-treated mice [78,95]. In patients with a recent myocardial infarction, the use of colchicine, an anti-inflammatory drug with NLRP3 inhibition activity [96], significantly lowered the risk (compared to the placebo) of ischemic cardiovascular events, such as death from cardiovascular causes, resuscitated cardiac arrest, myocardial infarction, stroke, and urgent hospitalization for angina leading to coronary revascularization [97]. On the other hand, the use of canakinumab, a monoclonal antibody that targets interleukin-1β, in a study involving 10,061 patients with previous myocardial infarction, showed reductions in hospitalizations for unstable angina that led to urgent revascularization in myocardial infraction, stroke, or death from any cause when compared to the placebo group [98]. Thus, unraveling the role of NLRP3 inflammasome in AMI holds promise for developing novel therapeutic strategies to attenuate myocardial damage and improve clinical outcomes in patients with acute coronary syndromes.

## 5. Role of NLRP3 Inflammasome Activity and Atrial Fibrillation

**Figure 2:**
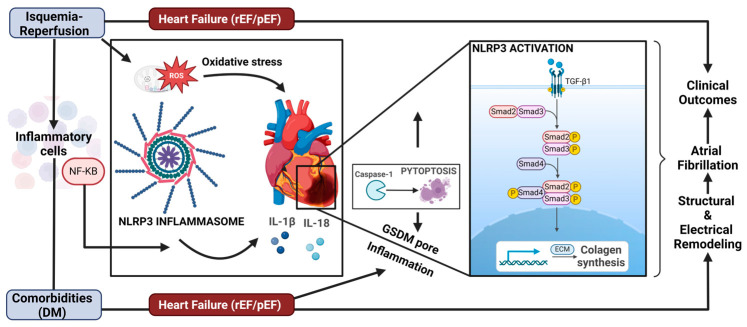
Indirect mechanisms of SGLT2i inhibition on the NLRP3 pathway and its effects on ventricular remodeling. The contribution of CV risk factors such as diabetes mellitus in heart failure (HFpEF) and the contribution of ischemia–reperfusion. The NLPR3 pathway determines the secretion of the cytokines IL-1beta and IL-18, which induce an increase in the extracellular matrix, ECM. In addition, collagen synthesis is increased, contributing to structural remodeling. Caspase-1 also cleaves Gasdermin-D (GSDM-D), which forms pores within the plasma membrane, driving a form of inflammatory cell death called pyroptosis. NF-KB; nuclear farctor KappaB; rEF, reduced ejection fraction; pEF, preserved ejection fraction; TGF-β1, transforming growth factor beta-1.

Experimental animal models have demonstrated that NLRP3 components are present in cardiomyocytes and cardiac fibroblasts. Recent protocols have identified the NLRP3 inflammasome as a crucial factor in the development of cardiomyopathies and atrial fibrillation (AF), suggesting a potential pathway for novel therapeutic agents [99]. An increased inflammatory profile is often linked to the onset of AF [100], with higher levels of circulating IL-1β and IL-18 positively correlating with the progression from paroxysmal AF (pAF) to persistent AF (perAF). Additionally, left atrial dilatation, an independent risk factor in regard to the induction of AF, has been observed in affected patients [101,102]. Recent findings indicate that the activity of the NLRP3 inflammasome is heightened in cardiomyocytes from patients with both pAF and perAF [103]. This inflammasome is expressed and upregulated in the non-immune cardiac cells of these patients, and its activity in human cardiomyocytes corresponds with the progression of AF to more persistent forms that lead to cardiac remodeling. In the case of POAF, recent data suggest that the cardiac anatomical or functional substrates determine which atria will cross the AF threshold, initiating POAF. In patients with an altered preoperative ventricular function, the inflammatory substrate is enhanced, accelerating electrical ventricular remodeling and re-entry [56]. A study involving the NLRP-3 pathways has provided further evidence linking inflammasome signaling in atrial myocytes to the development of arrhythmias. It found that this molecular pathway was sufficient for inducing arrhythmias and that markers of inflammasome activation were elevated in the atrial tissue of patients with AF [103,104]. Additionally, events that occur during extracorporeal circulation, along with postoperative inflammation, can worsen pre-existing calcium-homeostasis abnormalities. This can induce delayed afterdepolarization potential and lead to electrical remodeling, which may result in being predisposed to POAF [104]. Studies on rabbits utilizing ziprasidone, which induces atrial arrhythmia, including atrial fibrillation (AF), have shown increased activation of the NLRP3 inflammasome and ROS production via the PI3K/Akt/mTOR pathway [105]. Conversely, a study of salt-sensitive Dahl rats with heart failure with preserved ejection fraction (HFpEF) demonstrated that inhibition of the NLRP3 inflammasome with dapansutrile reduces atrial fibrillation (AF) by decreasing atrial inflammation and improving calcium handling [106]. Recent studies investigated the relationship between clonal hematopoiesis of indeterminate potential (CHIP), specifically TET methylcytosine dioxygenase 2 (TET2) CHIP, and atrial fibrillation (AF) in the UK Biobank, finding a modest association. Using mice with hematopoietic-specific Tet2 inactivation, they demonstrated that Nlrp3 inflammasome activation is implicated in atrial arrhythmogenesis, suggesting the therapeutic potential of NLRP3 inhibition in TET2 CHIP cases [107]. Therefore, the activation of NLRP3 inflammasome in myocardial tissue can be established to link the preoperative status of patients undergoing cardiac surgery, the probable cardiovascular outcomes, and the eventual remodeling events (Figure 2).

Research conducted in clinical and experimental settings has demonstrated that inflammation plays an essential role in ventricular remodeling. Specifically, the NLRP3 inflammasome has been extensively studied as a central effector in the inflammatory response [56,108,109]. For example, ventricular pressure overload increases Ca^2+^ influx, which triggers the activation of the NLRP3 inflammasome pathway in cardiomyocytes, which leads to the recruitment of macrophages, leading to myocardial fibrosis [110]. Furthermore, in some animal models of fibrosis, a redox imbalance leads to the activation of the NLRP3 inflammasome, promoting collagen deposition and amplifying the pro-inflammatory reaction cascade, which determines fibrosis through the NLRP3/IL-1β [111,112]. In IR injury, NLRP3 upregulation is induced by the cross-talk between the NLRP3 inflammasome and mitochondria, which increases ROS production and the oxidant products, perpetuating mitochondrial dysfunction (Mishra et al., 2021) [113]. In an experimental model of ischemia–reperfusion, the inhibition of NADPH oxidase may also have anti-inflammatory effects and reduce the infarct size effects, suppressing the expression of inflammasome proteins, including NLRP3-ASC, caspase-1, IL-1β, and IL-18 [114]. Therefore, determining the relationship between the NLRP3 inflammasome and VR induction in order to develop effective inhibitors and attenuate the progression of HFpEF and the worst outcome is a novel paradigm.

## 6. Preventive Therapies for Cardiovascular IR Injury and Remodeling

The cardiac remodeling process involves electrical and structural changes in the myocardium that determine worse clinical outcomes and mortality [115]. For example, post-myocardial injury mortality correlates with advancing age, regardless of infarct size, which may be related to a higher prevalence of ventricular hypertrophy but also to a decreased immunological response, scarring, and autophagy [116]. In the context of IR injury after cardiac surgery, long-term remodeling accelerates cardiomyocyte death through oxidative stress, inflammation, and fibrosis, thus leading to a decline in cardiac function.

Different potential preventive therapies have been identified that focus on the underlying pathophysiological mechanisms, particularly in experimental studies in fibrosis and cardiac remodeling. Cell death and mitochondrial dysfunction have been key targets of investigation. According to these mechanisms of IR injury, various types of pharmacological interventions have been implemented to block cell death pathways. Experimental protocols have demonstrated that cyclosporine and neuregulin-1 can reduce apoptosis. Additionally, necrostatin-1 not only attenuates apoptosis by inhibiting caspase-8 but also reduces necrosis by blocking calpain activity. Furthermore, modulating chaperones and the ubiquitin–proteasome system, which regulates protein degradation, can also determine a reduction in cell death [117]. Fibrosis has also been a focus of therapeutic interventions, with the inhibition of thrombospondin-1 and galectin-3 being associated with a decrease in collagen content. Similar effects have been reported with the administration of torsemide and metformin [118,119]. Additionally, the administration of CXL-1020, a nitroxyl donor, has been found to enhance the sensitivity of contractile proteins to calcium, leading to functional improvement and a reduction in hypertrophy.

Mitochondrial uncoupling has been identified as a cardioprotective strategy under oxidative stress conditions, such as diabetes, drug resistance in neoplasic and tumoral enviroment, IR injury, and aging. Mitchondrial uncoupling proteins (UCPs) reduce the efficiency of oxidative phosphorylation and play a role in controlling mitochondrial pro-oxidant imbalances [120]. There is strong evidence that UCP2 and UCP3, which are homologs of UCP1 and are induced in the heart, help protect against mitochondrial oxidative ROS burst both in vitro and in vivo [121]. Moreover, cardiovascular ischemic comorbidities, particularly clinical events of cardiac IR, lead to increased production of ROS and dysregulation of mitochondrial biogenesis in cardiomyocytes [122]. Therefore, it can be concluded that mitochondrial protection strategies are due to the reduction in cellular and tissue damage involved in the pathophysiology of IR injury and cardiovascular dysfunction [123,124].

Various trials have focused on demonstrating the benefits of antioxidants in attenuating acute ROS injury in terms of myocardial electrical remodeling after IR injury, such as after a cardiopulmonary bypass [28,31,125,126]. However, this clinical setting changes when focusing on ventricular remodeling events that occur over a long-term period and lead to detrimental effects on cardiac function. In regard to preconditioning pharmacological strategies and multiple naturally available antioxidants, it has been evaluated that the administration of polyphenols, omega 3 fatty acids, and antioxidant vitamins in IR animal models has been effective [26,126,127]. For example, resveratrol, which has been shown to have cardioprotective effects in various animal studies, could reduce cardiac inflammation and ventricular remodeling in an ischemia–reperfusion rat model [128,129]. Other antioxidants, such as statins and antioxidant vitamins, have limited their use as preventive therapies in ventricular remodeling due to the absense of effects on ventricular function or the lack of demonstrated beneficial outcomes in clinical trials [26,129].

As research on SGLT-2 CV advanced our knowledge of pathophysiology, the positive effects on the heart and vassels became clear. In this setting, empagliflozin has significantly reduced the primary endpoint, which includes CV death, nonfatal myocardial infarction, and nonfatal stroke, in patients with type 2 diabetes. Specifically, it resulted in a 38% decrease in CV deaths when added to standard diabetes treatment [130,131]. In addition to the previously expressed lowering of blood pressure and body weight, this improvement is likely attributed to osmotic diuresis, which lowers cardiac preload and helps alleviate symptoms of heart failure [132]. With respect to cardiac remodeling, experimental studies showed that empagliflozin improved diabetic myocardial structure and function, decreased myocardial oxidative stress, and ameliorated myocardial fibrosis [133]. Mechanistic studies have indicated that empagliflozin suppressed oxidative stress and fibrosis through inhibition of pro-fibrotic pathways, such as the transforming growth factor β/Smad pathway and activation of Nrf2/ARE signaling, among other elements [134,135]. Other SGLT-2 inhibitors, such as dapagliflozin, have similar cardioprotective properties against doxorubicin-induced cardiotoxicity. They accomplish this by decreasing pro-oxidant imbalances, mitochondrial dysfunction, fibrosis, hypertrophy, and inflammation through the PI3K/AKT/Nrf2 signaling pathway [136]. Studies with animals and echocardiography results indicated that rats treated with dapagliflozin showed improved heart performance. Regarding surgical procedures, ventricular reconstruction has been used to reverse cardiac remodeling in post-myocardial infarction patients with large LV aneurysms; however, residual LV remodeling and dysfunction remain postoperatively [137]. In this experimental model, it has been found that dapaglifozin attenuates residual cardiac remodeling after ventricular reconstruction by normalizing some cardiac- and metabolism-related hub genes [138].

Oxidative stress plays a critical role in the modulation of cardiac remodeling, affecting various mechanisms involved in this process [139]. Several antioxidants can attenuate cardiac remodeling that is associated with different injuries by reducing oxidative stress, mainly in animal models. These findings suggest that antioxidants, including SGLT-2is, could become part of a therapeutic strategy to addressing ventricular remodeling and postoperative AF occurrence while reinforcing the anti-remodeling properties of anti-inflammatories.

### 6.1. SGLT-2 Inhibitors: Pharmacological Properties

Gliflozins are small molecules that are C-glycoside analogs of the sodium–glucose transporter 1 (SGLT-1) and 2 (SGLT-2) inhibitor glycoside phlorizin [140,141], with a higher selectivity for SGLT-2. Their main mechanism of action is blocking the SGLT-2, which is highly expressed in the apical membrane of proximal tubules and allows for the reabsorption of 90% of glucose filtered in the glomerulus. By blocking SGLT-2, gliflozins reduce blood glucose levels and cause glucose to be excreted through urine, which is also beneficial for lowering body weight [142].

The structure of gliflozins consists of a glucose ring, a proximal benzene ring, a distal benzene ring, and a methylene bridge between these two benzene rings. Other structures can replaced with the distal benzene ring, and slight radical substitutions in the three rings can modify the potency and pharmacokinetics of gliflozins [143]. Several gliflozins have been tested for the treatment of diabetes mellitus, demonstrating beneficial effects on major cardiovascular events [144]. The FDA has approved canagliflozin, dapagliflozin, empagliflozin, and ertugliflozin, while ipragliflozin and tofogliflozin have been approved in Japan [144].

A summary of the clinical pharmacokinetics of gliflozins is shown in Table 1.

#### 6.1.1. Antioxidants and Anti-Inflammatory Effects

Recent data suggest that SGLT-2is have antioxidant properties that may be key to reducing cardiovascular death in clinical trials. Oxidative stress is known to contribute to the development and progression of atherosclerosis and diabetes complications, and it may result from either increased free-radical production, a reduction in antioxidative capacity, or a combination of both [157]. SGLT-2is have been shown to act as free-radical scavengers and boost the antioxidative system [158]. However, these effects have only been demonstrated in in vitro models in regard to cardiac and vascular IR injury. It is important to note that these drugs are considered to have direct antioxidant effects, while their glucose-lowering effects indirectly influence the redox state by decreasing the activity of pro-oxidant sources of reactive oxygen species (ROS) and improving mitochondrial function [159].

Research indicates that gliflozins may decrease ROS sources by NADPH oxidase activity inhibition through various mechanisms [160]. In a genetic mice model of type 2 diabetes mellitus, empagliflozin was shown an attenuation oxidative stress by inducing the nuclear factor erythroid 2 (Nrf2)/antioxidant responsive element (ARE) pathway while inhibiting the transforming growth factor beta (TGFβ)/Smad pathway [161]. A clinical work involving cardiac biopsies and superoxide quantification demonstrated that canagliflozin can reduce NADPH activation following cardiac surgery with extracorporeal circulation [162]. Specifically, the expression of sodium–glucose cotransporter 1 (SGLT1) in human atrial myocardium was positively correlated with the production of radical superoxide (O_2_^•−^) as well as with pro-fibrotic, pro-inflammatory, and wall stress mRNA levels. It has been reported that NADPH activation can be blocked by canagliflozin through AMP kinase (AMPK)/Rac1 signaling, which also protects nitric oxide synthase (NOS) coupling by maintaining tetrahydrobiopterin levels, providing protection from their oxidation in vivo and in vitro. Recently, studies have indicated that Canaglifozin can attenuate lipotoxicity in cardiomyocytes by regulating inflammation and ferroptosis by activating the AMPK pathway [163]. These studies provide a novel direction for myocardial lipotoxicity and ferroptosis in regard to the treatment of diabetic cardiomyopathy associated with oxidative stress occurrence [164,165].

##### Anti-Inflammatory Effects

In cardiac failure and IR injury, higher pro-inflammatory cytokines levels induce cross-talk with ROS production [166], particularly the interleukins, transforming growth factor-β, nuclear factor κB, monocyte chemoattractant protein-1 (MCP-1), and tumor necrosis factor-α, which determine ROS and RNS amplification and cross-talk production methods [167]. There is evidence that neurohumoral activation during heart failure modulates the immune cell system with both preserved and reduced ejection fractions. Indeed, these cells express angiotensin I receptors, adrenoceptors, and natriuretic peptide receptors that are influenced by this activation [168]. Ang II modulates macrophage polarization, promoting the M2 macrophage phenotype, and this stimulation can influence the lymphocyte Th1/Th2 balance. Activation of β-adrenergic receptors in monocytes inhibits ROS production [169], while brain natriuretic peptides in macrophages can stimulate ROS production, upregulate IL-10, and inhibit IL-12 and TNF-α release by dendritic cells; additionally, their circulating pool cells can predict the recurrence of decompensated heart failure [170,171]. Recent evidence suggests that SGLT2is are also associated with anti-inflammatory in vivo and in vitro effects. In this view, dapagliflozin significantly diminished collagen synthesis, induced anti-inflammatory macrophages, and reduced myofibroblast differentiation after cardiac ischemia in rats [172]. Higher levels of 10 (IL-10) anti-inflammatory cytokine are shown in the dapagliflozin group, while the control group only presens ischemia. Also, empagliflozin reduced the human fibroblast activation and proliferation due to transforming the growth factor β1 (TGFβ1) pathway inhibition in a concentration-dependent manner while also reducing pro-fibrotic mediators [173]. Experimental studies with dapaglifozin show induction of cardioprotective mechanisms against injury-related ferroptosis using a rat model and in H9C2 cardiomyocytes subjected to hypoxia/reoxygenation (H/R)-model. Dapagliflozin reduced myocardial injury, mitigated reperfusion arrhythmia, and enhanced cardiac function. This was evidenced by a reduction in cardiac injury biomarkers, including troponin and B-natriuretic peptide, as well as improvements in pathological settings. It also prevented H/R-induced loss of cell viability in vitro [174].

An NLRP3 inhibitor is a drug that is capable of blocking the polymerization of the NLRP3 inflammasome and, therefore, preventing its activation [64]. A small-molecule structure exerts effects that block the pro-inflammatory effect on NLRP3 inflammasome activation in cell studies, such as MCC950, β-hydroxybutyrate, Bay 11-7082, dimethyl sulfoxide, and interferon type I. Therefore, these inhibitors demonstrate low efficacy and nonspecific actions. For inhibitors that block the IL-1β pathway, it should be noted that IL-1β secretion and extracellular activity are not the determined by NLRP3 inflammasome activation; moreover, other pro-inflammatory mediators, including HMGB1 and IL-18, may have some role in the pathophysiology of these diseases [175]. Also, while several NLRP3 inhibitors have been discovered (MCC950, Tranilast, OLT1177, etc.), SGLT2is do not directly inhibit NLRP3 aggregation. Table 2 shows the NLRP3 Inflammasome Inhibitors and potential mechanisms.

The cardioprotective mechanisms of SGLT-2is have been explored, including the reduction in cardiac and endothelial inflammation, as well as the inhibition of pro-oxidant damage. These effects can improve cardiac structure and function. However, the impact of SGLT-2is on pathophysiological mechanisms has not yet been elucidated. Currently, there are no clinical trials that explore the probable effects of SGLT-2is on cardiac IR during cardiac technical events, such as surgery or percutaneous coronary intervention procedures. In ongoing trials involving cardiac failure patients, SGLT-2i administration is associated with reduced systemic inflammation. Reviews involving both systematic and meta-analysis methodology in animals indicated that the use of SGLT-2is leads to lower the levels of IL-6, CRP, TNF-alpha, and MCP-1. Additionally, SGLT-2is have been shown to significantly suppress the activation of the NLRP3 inflammasome, which in turn reduces IL-1 beta secretion in human macrophages. SGLT-2i diminish epicardial fat, a source of pro-inflammatory adipokines, but this aspect has no relation to their glycemic effects [185]. In a recent trial of dapagliflozin vs. a placebo in diabetic individuals with left ventricular hypertrophy, 12 months of treatment with dapagliflozin caused a significant reduction in CRP [186]. Recently, genetically modified SGLT-2 inhibition was associated with a reduced risk of developing HF. Oro-inflammatory cytokines such as C-X-C motif chemokine ligand 10 (CXCL10) and the leukemia inhibitory factor were mechanistically related to both SGLT-2 inhibition and HF progression. A multivariate statistical analysis revealed that CXCL10 was the primary cytokine mediator related to HF [187]. In regard to POAF, the clinical evidence indicates that the inhibition of SGLT2is on this arrhythmia is not direct, as a few related-mechanisms are involved. In this view, various observational and clinical studies retrospectively show a lower AF occurrence in HF patients. The SGLT2i efficacy is not evident, and the randomized or Phase III trials present mixed results, with few demonstrating benefits and endpoints of statistical significance. Methodologically, the differences in study design, the variable population settings, and the follow-up duration do not support the solid evidence regarding SGLT2is as antiarrhythmic therapy in HF or postoperative settings, only indicating a reduction in ventricular arrhythmias [Stachteas et al., 2024, Zarei et al., 2024] [188,189]. Ongoing clinical studies have a probable outcome in regard to preventing POAF in patients subjected to coronary artery bypass graft surgery [Aghakouchakzadeh, 2024] [190].

In summary of some clinical and mechanistical evidence, SGLT2 inhibition and the derived physiological pathways may attenuate pro-oxidant damage partly by suppressing inflammatory mechanisms in classical and novel pathways [191]. In addition, neurohormonal-immune cross-talks responses are associated with the phenotype of cardiac remodeling and are derived to either favorable or maladaptive functional and structural responses. However, the clinical effects on postoperative and CPB settings are not well characterized.

#### 6.1.2. SGLT2is and Ventricular Remodeling

Current pharmacological therapies to prevent damage caused by myocardial IR and subsequent ventricular remodeling have been suboptimal. These treatments mainly target events around the time of surgery or factors such as oxidative stress and tissue inflammation. Therefore, therapies focused solely on antioxidants and/or anti-inflammatory agents around the time of surgery, to address issues like postoperative atrial fibrillation (POAF) or ventricular function, may not be as clinically effective as hoped. Clinical trials have shown this, for example, in the case of POAF [6,192]. Additionally, in patients with normal heart rhythms undergoing heart surgery, changes in the right atrial appendage tissue do not predict the occurrence of POAF [193].

Recently, SGLT-2is have been linked to significant reductions in cardiovascular death and HF hospitalization in people with type 2 diabetes mellitus [194,195]. The cardioprotective effects of these drugs are believed to be related to their impact on kidney function, induction of glycosuria, and inhibition of pro-inflammatory pathways at the tissue level [195,196]. In diabetic patients, hyperglycemia and the pro-inflammatory status induced by insulin resistance play important roles in impaired cardiac function. Therefore, SGLT-2i drugs have multiple cardioprotective mechanisms against HF. These include lowering intracellular sodium levels, which is well known to be cardioprotective by preventing oxidative stress and subsequent cardiomyocyte death [197]. Other cardioprotective mechanisms include reducing cardiac fibrosis, decreasing collagen synthesis, and inhibiting the differentiation of myofibroblasts and pro-fibrotic markers seen in animal models of heart remodeling after a myocardial infarction [172,198]. Preclinical studies also suggest that specific SGLT-2i drugs may improve HF outcomes in non-diabetic patients through direct anti-inflammatory effects on the heart. For example, preserved cardiac function by empagliflozin is associated with reduced cardiac expression of pro-inflammatory cytokines as well as lower macrophage infiltration mediated by the NLRP3 inflammasome [199]. In terms of clinical evidence, several trials have demonstrated that SGLT-2i drugs have a cardioprotective effect and can reduce the hemodynamic effects of HF, ultimately lowering cardiovascular mortality. However, the effects and mechanisms of SGLT-2is on ventricular remodeling are still largely unknown [200,201]. Finally, in the case of HFpEF patients, a specific trial (EMPEROR-Preserved Trial) enrolled ≈ 5750 individuals with preserved HF (ejection fraction > 40%) with and without type 2 diabetes who were patients that were assigned a placebo or empagliflozin 10 mg/day for two years, adding to the research on pharmacological standard therapy for HFpEF and cardiovascular clinical conditions [202]. This trial showed a reduction in mortality and HF hospitalization in the those taking empaglifozin compared to the placebo group, regardless of diabetes status. Currently, another trial (EMPA-VISION) is currently assessing the effects of empagliflozin treatment on cardiac energy metabolism in human subjects in vivo using cMRI, complementing the results of the previous follow-up in patients with HFpEF [203]. However, these trials have not fully explored the pathophysiological and molecular aspects.

## 7. Concluding Remarks

The role of NLRP3 inflammasome signaling pathways and the SGLT-2 cotransporter in inflammatory and oxidative stress-induced processes related to various cardiovascular diseases is becoming increasingly clear and compelling. Since the inhibition of the SGLT-2 cotransporter modulates the NLRP3 inflammasome, at least in cardiovascular diseases associated with type 2 diabetes, it is suggested that this approach may also help to protect the heart from adverse pathological ventricular remodeling and cardiac arrhythmias, including postoperative atrial fibrillation, by suppressing inflammasome activity [Kim et al., 2020] [204]. However, the direct or indirect effects of NLRP3 activity inhibition on both POAF and cardiac remodeling have not yet been evaluated in regard to clinical cardiac postoperative settings and should therefore only be considered hypothetically. Nevertheless, given its potential benefits—especially in patients with heart failure with preserved ejection fraction, where therapeutic options are limited—it is essential to explore this approach clinically.

## Figures and Tables

**Table 1:** Summary of the clinical pharmacokinetics parameters of gliflozins.

Drug	Dose	T_max_	Half-Life (t_1/2_)	References
Canagliflozin	25–400 mg	1.0–4.0 h	10.22–16.30 h	[145,146]
Dapagliflozin	25–50 mg	0.5–1.5 h	8.1–16.4 h	[147,148]
Empagliflozin	1–100 mg	1.0–2.5 h	6.1–16.5 h	[149,150]
Ertugliflozin	5–25 mg	1.0–4.0 h	9.91–16.87 h	[151,152]
Ipragliflozin	25–100 mg	0.5–0.9 h	10.5–11.3	[153,154]
Tofogliflozin	10–640 mg	0.5–2.0 h	3.81–6.21 h	[155,156]

**Table 2:** NLRP3 Inflammasome Inhibitors, mechanisms, and effects regarding SGLT2.

Inhibitor	Mechanism of Inhibition	Effect on SGLT2	Reference
**MCC950**	Blocks NLRP3 activation by inhibiting ATPase activity, preventing inflammasome assembly	No effect on SGLT2 identified	[176]
**Glyburide**	Inhibits NLRP3 activation through K+ efflux blockade	No effect on SGLT2 identified	[177]
**Oridonin**	Covalently binds to NLRP3, disrupting inflammasome assembly	No effect on SGLT2 identified	[178]
**OLT1177 (Dapansutrile)**	Inhibits ASC oligomerization, a key step in inflammasome activation	No effect on SGLT2 identified	[179]
**CY-09**	Binds to NACHT domain, blocking NLRP3 ATPase activity	Inhibits NLRP3 oligomerization by binding NACHT domain	[180]
**Tranilast**	Inhibits NLRP3 oligomerization by binding NACHT domain	No effect on SGLT2 identified	[181,182]
**beta-hydroxybutyrate (β-OHB)**	Inhibits oxidative stress and it can protect the function of mitochondria and exert an anti-inflammatory effect, as it is an endogenous NLRP3 inflammasome inhibitor.	An increase in β-OHB levels by the administration of SGLT-2 inhibitors	[183]
**SGLT2 inhibitors (e.g., Empagliflozin)**	Reduce NLRP3 activation indirectly by suppressing NF-κB signaling and promoting autophagy	Yes, as primary function	[184]

NLRP3, NOD-, LRR- and pyrin domain-containing protein 3; NACHT, nucleoside triphosphatase (NTPase) domain; NF-κB, nuclear factor-kappa B.

## References

[1] Axtell A.L., Moonsamy P., Melnitchouk S., Tolis G., Jassar A.S., D’Alessandro D.A., Villavicencio M., Cameron D.E., Sundt T.M. (2020). Preoperative predictors of new-onset prolonged atrial fibrillation after surgical aortic valve replacement. J. Thorac. Cardiovasc. Surg..

[2] Weymann A., Popov A.F., Sabashnikov A., Ali-Hasan-Al-Saegh S., Ryazanov M., Tse G., Mirhosseini S.J., Liu T., Lotfaliani M., Sedaghat M. (2018). Baseline and postoperative levels of C-reactive protein and interleukins as inflammatory predictors of atrial fibrillation following cardiac surgery: A systematic review and meta-analysis. Kardiol. Pol..

[3] Tadic M., Ivanovic B., Zivkovic N. (2011). Predictors of atrial fibrillation following coronary artery bypass surgery. Med. Sci. Monit..

[4] Nair S.G. (2010). Atrial fibrillation after cardiac surgery. Ann. Card. Anaesth..

[5] Dobrev D., Aguilar M., Heijman J., Guichard J.B., Nattel S. (2019). Postoperative atrial fibrillation: Mechanisms, manifestations and management. Nat. Rev. Cardiol..

[6] Liu Y., Wu F., Wu Y., Elliott M., Zhou W., Deng Y., Ren D., Zhao H. (2021). Mechanism of IL-6-related spontaneous atrial fibrillation after coronary artery grafting surgery: IL-6 knockout mouse study and human observation. Transl. Res..

[7] Topkara V., Cheema F.H., Kesavaramanujam S., Mercando M.L., Cheema A.F., Namerow P.B., Argenziano M., Naka Y., Oz M.C., Esrig B.C. (2005). Coronary artery bypass grafting in patients with low ejection fraction. Circulation.

[8] Dogan S.M., Buyukates M., Kandemir O., Aydin M., Gursurer M., Acikgoz S., Yavuzer R., Cam F., Dursun A. (2007). Predictors of atrial fibrillation after coronary artery bypass surgery. Coron. Artery Dis..

[9] Mathis M.R., Duggal N.M., Janda A.M., Fennema J.L., Yang B.O., Pagani F.D., Maile M.D., Hofer R.E., Jewell E.S., Engoren M.C. (2021). Reduced Echocardiographic Inotropy Index after Cardiopulmonary Bypass Is Associated with Complications After Cardiac Surgery: An Institutional Outcomes Study. J. Cardiothorac. Vasc. Anesth..

[10] Russo C., Jin Z., Sera F., Lee S.E., Homma S., Rundek T., Elkind M.S., Sacco R.L., Di Tullio M.R. (2015). Left Ventricular Systolic Dysfunction by Longitudinal Strain Is an Independent Predictor of Incident Atrial Fibrillation: A Community-Based Cohort Study. Circ. Cardiovasc. Imaging.

[11] Thavendiranathan P., Negishi T., Somerset E., Negishi E., Penicka M., Lemieux J., Aakhus S., Miyazaki S., Shirazi M., Galderisi M. (2021). Strain-Guided Management of Potentially Cardiotoxic Cancer Therapy. J. Am. Coll. Cardiol..

[12] Kawczynski M., Gilbers M., Walle S., Schalla S., Crijns H.J., Maessen J.G., Schotten U., Maesen B., Bidar E. (2021). Role of pre-operative transthoracic echocardiography in predicting post-operative atrial fibrillation after cardiac surgery: A systematic review of the literature and meta-analysis. Europace.

[13] Park J.J., Park J.B., Park J.H., Cho G.Y. (2018). Global longitudinal strain to predict mortality in patients with acute heart failure. J. Am. Coll. Cardiol..

[14] Kalam K., Otahal P., Marwick T.H. (2014). Prognostic implications of global LV dysfunction: A systematic review and meta-analysis of global longitudinal strain and ejection fraction. Heart.

[15] Stampehl M.R., Mann D.L., Nguyen J.S., Cota F., Colmenares C., Dokainish H. (2015). Speckle strain echocardiography predicts outcome in patients with heart failure with both depressed and preserved left ventricular ejection fraction. Echocardiography.

[16] Wang C.H., Chan Y., Chien-Chia V., Lee H.-F., Hsiao F., Chu P.-H. (2021). Incremental prognostic value of global myocardial work over ejection fraction and global longitudinal strain in patients with heart failure and reduced ejection fraction. Eur. Heart J. Cardiovasc. Imaging.

[17] Buggey J., Alenezi F., Yoon H.J., Phelan M., DeVore A.D., Khouri M.G., Schulte P.J., Velazquez E.J. (2017). Left ventricular global longitudinal strain in patients with heart failure with preserved ejection fraction: Outcomes following an acute heart failure hospitalization. ESC Heart Fail..

[18] Mihm M., Yu F., Carnes C., Reiser P.J., McCarthy P.M., Van Wagoner D.R., Bauer J.A. (2001). Impaired Myofibrillar Energetics and Oxidative Injury During Human Atrial Fibrillation. Circulation.

[19] Carnes C., Chung M., Nakayama T., Nakayama H., Baliga R.S., Piao S., Kanderian A., Pavia S., Hamlin R.L., McCarthy P.M. (2001). Ascorbate attenuates atrial pacing-induced peroxynitrite formation and electrical remodeling and decreases the incidence of postoperative atrial fibrillation. Circ. Res..

[20] Chang J., Chen M., Liu W., Yang C., Chen C., Chen Y.L., Pan K., Tsai T., Chang H. (2011). Atrial myocardial nox2 containing NADPH oxidase activity contribution to oxidative stress in mitral regurgitation: Potential mechanism for atrial remodeling. Cardiovasc. Pathol..

[21] Mighiu A., Recalde A., Ziberna K., Carnicer R., Tomek J., Bub G., Brewer A.C., Verheule S., Shah A.M., Simon J.M. (2021). Inducibility, but not stability, of atrial fibrillation is increased by NOX2 overexpression in mice. Cardiovasc. Res..

[22] Hansen M., Sadredini M., Hasic A., Eriksen M., Stokke M.K. (2023). Myocardial oxidative stress is increased in early reperfusion, but systemic antioxidative therapy does not prevent ischemia reperfusion arrhythmias in pigs. Front. Cardiovasc. Med..

[23] Qin Y., Hoek V.T., Wojcik K., Anderson T., Li C., Shao Z., Becker L.B., Hamann K.J. (2004). Caspase-dependent cytochrome c release and cell death in chick cardiomyocytes after simulated ischemia-reperfusion. Am. J. Physiol. Heart Circ. Physio..

[24] Martins D., Garcia L.R., Queiroz D.A., Lazzarin T., Rodrigues C., da Silva P., Polegato B.F., de Paiva S.A.R., Azevedo P.S., Minicucci M.F. (2022). Oxidative Stress as a Therapeutic Target of Cardiac Remodeling. Antioxidants.

[25] Jayaram R., Jones M., Reilly S., Crabtree M., Pal N., Goodfellow N., Nahar K., Simon J., Carnicer R., DeSilva R. (2022). Atrial nitroso-redox balance and refractoriness following on-pump cardiac surgery: A randomized trial of atorvastatin. Cardiovasc. Res..

[26] Farias J.G., Molina V.M., Carrasco R., Zepeda A.B., Figueroa E., Letelier P., Castillo R.L. (2017). Antioxidant Therapeutic Strategies for Cardiovascular Conditions Associated with Oxidative Stress. Nutrients.

[27] Kazzi M.E., Rayner B., Chami B., Dennis J.M., Thomas S. (2020). Witting PK Neutrophil-Mediated Cardiac Damage After Acute Myocardial Infarction: Significance of Defining a New Target Cell Type for Developing Cardioprotective Drugs. Antioxid. Redox Signal..

[28] Castillo R.L., Rodrigo R., Pérez F., Cereceda M., Asenjo R., Zamorano J., Navarrete R., Villalabeitia E., Sanz J., Baeza C. (2011). Antioxidant therapy reduces oxidative and inflammatory tissue damage in patients subjected to cardiac surgery with extracorporeal circulation. Basic Clin. Pharmacol. Toxicol..

[29] Sánchez F.J., Gonzalez V.A., Farrando M., Jayat A.O., Segovia-Roldan M., García-Mendívil L., Ordovás L., Prado N.J., Pueyo E., Diez E.R. (2020). Atrial Dyssynchrony Measured by Strain Echocardiography as a Marker of Proarrhythmic Remodeling and Oxidative Stress in Cardiac Surgery Patients. Oxid. Med. Cell Longev..

[30] Kim Y.M., Kattach H., Ratnatunga C., Pillai R., Channon K.M., Casadei B. (2008). Association of atrial nicotinamide adenine dinucleotide phosphate oxidase activity with the development of atrial fibrillation after cardiac surgery. J. Am. Coll. Cardiol..

[31] Rodrigo R., Korantzopoulos P., Cereceda M., Asenjo R., Zamorano J., Villalabeitia E., Baeza C., Aguayo R., Castillo R., Carrasco R. (2013). A randomized controlled trial to prevent postoperative atrial fibrillation by antioxidant reinforcement. J. Am. Coll. Cardiol..

[32] Wu J., Marchioli R., Silletta M., Masson S., Sellke F.W., Libby P., Milne G.L., Brown N.J., Lombardi F., Damiano R.J. (2015). Oxidative Stress Biomarkers and Incidence of Postoperative Atrial Fibrillation in the Omega-3 Fatty Acids for Prevention of Postoperative Atrial Fibrillation (OPERA) Trial. J. Am. Heart Assoc..

[33] Yan X., Anzai A., Katsumata Y., Matsuhashi T., Ito K., Endo J., Yamamoto T., Takeshima A., Shinmura K., Shen W. (2013). Temporal dynamics of cardiac immune cell accumulation following acute myocardial infarction. J. Mol. Cell Cardiol..

[34] Berezin A.E., Berezin A.A. (2020). Adverse Cardiac Remodelling after Acute Myocardial Infarction: Old and New Biomarkers. Dis. Markers.

[35] van den Berge J.C., Vroegindewey M.M., Veenis J.F., Brugts J.J., Caliskan K., Manintveld O.C., Akkerhuis K.M., Boersma E., Deckers J.W., Constantinescu A.A. (2021). Left ventricular remodelling and prognosis after discharge in new-onset acute heart failure with reduced ejection fraction. ESC Heart Fail..

[36] Lazzerini P., Abbate A., Boutjdir M., Capecchi P.L. (2023). Fir(e)ing the Rhythm Inflammatory Cytokines and Cardiac Arrhythmias. JACC Basic Transl. Sci..

[37] Lamm G., Auer J., Weber T., Berent R., Ng C., Eber B. (2006). Postoperative white blood cell count predicts atrial fibrillation after cardiac surgery. J. Cardiothorac. Vasc. Anesth..

[38] Fontes M.L., Amar D., Kulak A., Koval K., Zhang H., Shi W. (2009). Increased preoperative white blood cell count predicts postoperative atrial fibrillation after coronary artery bypass surgery. J. Cardiothorac. Vasc. Anesth..

[39] Friedrichs K., Adam M., Remane L. (2014). Induction of atrial fibrillation by neutrophils critically depends on CD11b/CD18 integrins. PLoS ONE.

[40] Erdolu B., Kagan A., Engin M. (2020). The Relationship between the HATCH Score, Neutrophil to Lymphocyte Ratio and Postoperative Atrial Fibrillation After Off-Pump Coronary Artery Bypass Graft Surgery. Heart Surg. Forum..

[41] Wu N., Xu B., Xiang Y., Wu L., Zhang Y., Ma X., Tong S., Shu M., Song Z., Li Y. (2013). Association of inflammatory factors with occurrence and recurrence of atrial fibrillation: A meta-analysis. Int. J. Cardiol..

[42] Ward C.A., Bazzazi H., Clark R.B., Nygren A., Giles W.R. (2006). Actions of emigrated neutrophils on Na(+) and K(+) currents in rat ventricular myocytes. Prog. Biophys. Mol. Biol..

[43] Holzwirth E., Kornej J., Erbs S., Obradovic D., Bollmann A., Hindricks G., Thiele H., Büttner P. (2020). Myeloperoxidase in atrial fibrillation: Association with progression, origin and influence of renin-angiotensin system antagonists. Clin. Res. Cardiol..

[44] Wu Q., Liu H., Liao J., Zhao N., Tse G., Han B., Chen L., Huang Z., Du Y. (2020). Colchicine prevents atrial fibrillation promotion by inhibiting IL-1β-induced IL-6 release and atrial fibrosis in the rat sterile pericarditis model. Biomed. Pharmacother..

[45] Scott L., Fender L., Saljic A., Li L., Chen X., Wang X., Linz D., Lang J., Hohl M., Twomey D. (2021). NLRP3 inflammasome is a key driver of obesity-induced atrial arrhythmias. Cardiovasc. Res..

[46] Hemenway G., Frishman W.H. (2021). Therapeutic Implications of NLRP3-Mediated Inflammation in Coronary Artery Disease. Cardiol. Rev..

[47] Shateri H., Manafi B., Tayebinia H., Karimi J., Khodadadi I. (2021). Imbalance in thioredoxin system activates NLRP3 inflammasome pathway in epicardial adipose tissue of patients with coronary artery disease. Mol. Biol. Rep..

[48] Parent S., Vaka R., Amant J., Kahn S., Van Remortel S., Bi C., Courtman D., Stewart D.J., Raymond D., Davis D.R. (2024). Inactivation of the NLRP3 inflammasome mediates exosome-based prevention of atrial fibrillation. Theranostics.

[49] Parent S., Amant J., Van Remortel S., Kahn S., Vaka R., Courtman D., Stewart D.J., Davis D.R. (2024). Atrial Fibrosis and Inflammation in Postoperative Atrial Fibrillation: Comparative Effects of Amiodarone, Colchicine, or Exosomes. JACC Clin. Electrophysiol..

[50] Chau Y., Yoo J.W., Yuen H. (2021). The impact of post-operative atrial fibrillation on outcomes in coronary artery bypass graft and combined procedures. J. Geriatr. Cardiol..

[51] Kuppahally S., Akoum N., Burgon N.S. (2010). Left atrial strain and strain rate in patients with paroxysmal and persistent atrial fibrillation: Relationship to left atrial structural remodeling detected by delayed-enhancement MRI. Circ. Cardiovasc. Imaging.

[52] Gopinathannair R., Chen L.Y., Chung M.K., Cornwell W.K., Furie K.L., Lakkireddy D.R., Marrouche N.F., Natale A., Olshansky B., Joglar J.A. (2021). Managing Atrial Fibrillation in Patients With Heart Failure and Reduced Ejection Fraction: A Scientific Statement From the American Heart Association. Circ. Arrhythmia Electrophysiol..

[53] Badheka A.O., Shah N., Grover P.M., Patel N.J., Chothani A., Mehta K., Singh V., Deshmukh A., Savani G.T., Rathod A. (2014). Outcomes in atrial fibrillation patients with and without left ventricular hypertrophy when treated with a lenient rate-control or rhythm-control strategy. Am. J. Cardiol..

[54] Packer M. (2020). HFpEF Is the Substrate for Stroke in Obesity and Diabetes Independent of Atrial Fibrillation. JACC Heart Fail..

[55] Wang S., Yuan Y.-H., Chen N.-H., Wang H.-B. (2019). The mechanisms of NLRP3 inflammasome/pyroptosis activation and their role in Parkinson’s disease. Int. Immunopharmacol..

[56] Yan Z., Qi Z., Yang X., Ji N., Wang Y., Shi Q., Li M., Zhang J., Zhu Y. (2021). The NLRP3 inflammasome: Multiple activation pathways and its role in primary cells during ventricular remodeling. J. Cell Physiol..

[57] Elliott E.I., Sutterwala F.S. (2015). Initiation and perpetuation of NLRP 3 inflammasome activation and assembly. Immunol. Rev..

[58] Noma A. (1983). ATP-regulated K+ channels in cardiac muscle. Nature.

[59] Rodrigo G.C. (1993). The Na^+^-dependence of Na^+^-activated K^+^-channels (I_K(Na)_) in guinea pig ventricular myocytes, is different in excised inside/out patches and cell-attached patches. Pflügers Arch..

[60] Carmeliet E. (1999). Cardiac ionic currents and acute ischemia: From channels to arrhythmias. Physiol. Rev..

[61] Mitani A., Shattock M., Physiology C. (1992). Role of Na-activated K channel, Na-K-Cl cotransport, and NaK pump in [K] e changes during ischemia in rat heart. Am. J. Physiol. Heart Circ. Physiol..

[62] Shen S., Wang Z., Sun H., Ma L. (2022). Role of NLRP3 Inflammasome in Myocardial Ischemia-Reperfusion Injury and Ventricular Remodeling. Med. Sci. Monit..

[63] Amano F., Akamatsu Y. (1991). A lipopolysaccharide (LPS)-resistant mutant isolated from a macrophage-like cell line, J774. 1, exhibits an altered activated-macrophage phenotype in response to LPS. Infect. Immun..

[64] Yang Y., Wang H., Kouadir M., Shi F. (2019). Recent advances in the mechanisms of NLRP3 inflammasome activation and its inhibitors. Cell Death Dis..

[65] Gong T., Yang Y., Jin T., Jiang W., Zhou R. (2018). Orchestration of NLRP3 inflammasome activation by ion fluxes. Trends Immunol..

[66] Murphy E., Steenbergen C. (2008). Mechanisms underlying acute protection from cardiac ischemia-reperfusion injury. Physiol. Rev..

[67] Wyss-Coray T., Mucke L. (2002). Inflammation in neurodegenerative disease—A double-edged sword. Neuron.

[68] Fioranelli M., Roccia M.G., Flavin D., Cota L. (2021). Regulation of Inflammatory Reaction in Health and Disease. Int. J. Mol. Sci..

[69] Jorquera G., Russell J., Monsalves-Álvarez M., Cruz G., Valladares-Ide D., Basualto-Alarcón C., Barrientos G., Estrada M., Llanos P. (2021). NLRP3 Inflammasome: Potential Role in Obesity Related Low-Grade Inflammation and Insulin Resistance in Skeletal Muscle. Int. J. Mol. Sci..

[70] Danesh J., Whincup P., Walker M., Lennon L., Thomson A., Appleby P., Gallimore J.R., Pepys M.B. (2000). Low grade inflammation and coronary heart disease: Prospective study and updated meta-analyses. BMJ.

[71] Zheng D., Liwinski T., Elinav E. (2020). Inflammasome activation and regulation: Toward a better understanding of complex mechanisms. Cell Discov..

[72] Cordero M.D., Alcocer-Gómez E., Ryffel B. (2018). Gain of function mutation and inflammasome driven diseases in human and mouse models. J. Autoimmun..

[73] He Y., Hara H., Núñez G. (2016). Mechanism and Regulation of NLRP3 Inflammasome Activation. Trends Biochem. Sci..

[74] Schroder K., Tschopp J. (2010). The inflammasomes. Cell.

[75] Lu A., Wu H. (2015). Structural mechanisms of inflammasome assembly. FEBS J..

[76] Masumoto J., Taniguchi S., Ayukawa K., Sarvotham H., Kishino T., Niikawa N., Hidaka E., Katsuyama T., Higuchi T., Sagara J. (1999). ASC, a novel 22-kDa protein, aggregates during apoptosis of human promyelocytic leukemia HL-60 cells. J. Biol. Chem..

[77] Swanson K.V., Deng M., Ting J. (2019). The NLRP3 inflammasome: Molecular activation and regulation to therapeutics. Nat. Rev. Immunol..

[78] Toldo S., Mezzaroma E., Buckley L.F., Potere N., Di Nisio M., Biondi-Zoccai G., Van Tassell B., Abbate A. (2022). Targeting the NLRP3 inflammasome in cardiovascular diseases. Pharmacol. Ther..

[79] Bai B., Yang Y., Wang Q., Li M., Tian C., Liu Y., Aung L.H., Li P., Yu T., Chu X. (2020). NLRP3 inflammasome in endothelial dysfunction. Cell Death Dis..

[80] Li X., Zhang Z., Luo M., Cheng Z., Wang R., Liu Q., Lv D., Yan J., Shang F., Luo S. (2022). NLRP3 inflammasome contributes to endothelial dysfunction in angiotensin II-induced hypertension in mice. Microvasc. Res..

[81] Sun H., Ren X., Xiong X., Chen Y., Zhao M., Wang J., Zhou Y., Han Y., Chen Q., Li Y. (2017). NLRP3 inflammasome activation contributes to VSMC phenotypic transformation and proliferation in hypertension. Cell Death Dis..

[82] Wang R., Wang Y., Mu N., Lou X., Li W., Chen Y., Fan D., Tan H. (2017). Activation of NLRP3 inflammasomes contributes to hyperhomocysteinemia-aggravated inflammation and atherosclerosis in apoE-deficient mice. Lab. Invest..

[83] Toldo S., Abbate A. (2018). The NLRP3 inflammasome in acute myocardial infarction. Nat. Rev. Cardiol..

[84] Krishnan S.M., Ling Y.H., Huuskes B.M., Ferens D.M., Saini N., Chan C.T., Diep H., Kett M.M., Samuel C.S., Kemp-Harper B.K. (2019). Pharmacological inhibition of the NLRP3 inflammasome reduces blood pressure, renal damage, and dysfunction in salt-sensitive hypertension. Cardiovasc. Res..

[85] Duewell P., Kono H., Rayner K.J., Sirois C.M., Vladimer G., Bauernfeind F.G., Abela G.S., Franchi L., Nuñez G., Schnurr M. (2010). NLRP3 inflammasomes are required for atherogenesis and activated by cholesterol crystals. Nature.

[86] Usui F., Shirasuna K., Kimura H., Tatsumi K., Kawashima A., Karasawa T., Hida S., Sagara J., Taniguchi S., Takahashi M. (2012). Critical role of caspase-1 in vascular inflammation and development of atherosclerosis in Western diet-fed apolipoprotein E-deficient mice. Biochem. Biophys. Res. Commun..

[87] Menu P., Pellegrin M., Aubert J.F., Bouzourene K., Tardivel A., Mazzolai L., Tschopp J. (2011). Atherosclerosis in ApoE-deficient mice progresses independently of the NLRP3 inflammasome. Cell Death Dis..

[88] Zheng Y., Xu L., Dong N., Li F. (2022). NLRP3 inflammasome: The rising star in cardiovascular diseases. Front. Cardiovasc. Med..

[89] Varghese G.P., Folkersen L., Strawbridge R.J., Halvorsen B., Yndestad A., Ranheim T., Krohg-Sørensen K., Skjelland M., Espevik T., Aukrust P. (2016). NLRP3 Inflammasome Expression and Activation in Human Atherosclerosis. J. Am. Heart Assoc..

[90] Toldo S., Mezzaroma E., Mauro A.G., Salloum F., Van Tassell B.W., Abbate A. (2015). The inflammasome in myocardial injury and cardiac remodeling. Antioxid. Redox Signal..

[91] Toldo S., Mezzaroma E., McGeough M.D., Peña C.A., Marchetti C., Sonnino C., Van Tassell B.W., Salloum F.N., Voelkel N.F., Hoffman H.M. (2015). Independent roles of the priming and the triggering of the NLRP3 inflammasome in the heart. Cardiovasc Res..

[92] Higashikuni Y., Liu W., Numata G., Tanaka K., Fukuda D., Tanaka Y., Hirata Y., Imamura T., Takimoto E., Komuro I. (2023). NLRP3 Inflammasome Activation Through Heart-Brain Interaction Initiates Cardiac Inflammation and Hypertrophy During Pressure Overload. Circulation.

[93] Van Tassell B.W., Trankle C.R., Canada J.M., Carbone S., Buckley L., Kadariya D., Del Buono M.G., Billingsley H., Wohlford G., Viscusi M. (2018). IL-1 Blockade in Patients with Heart Failure with Preserved Ejection Fraction. Circ. Heart Fail..

[94] Kawaguchi M., Takahashi M., Hata T., Kashima Y., Usui F., Morimoto H., Izawa A., Takahashi Y., Masumoto J., Koyama J. (2011). Inflammasome activation of cardiac fibroblasts is essential for myocardial ischemia/reperfusion injury. Circulation.

[95] Gao R., Li X., Xiang H., Yang H., Lv C., Sun X., Chen H., Gao Y., Yang J., Luo W. (2021). The covalent NLRP3-inflammasome inhibitor Oridonin relieves myocardial infarction induced myocardial fibrosis and cardiac remodeling in mice. Int. Immunopharmacol..

[96] Zhang Q., Wang L., Wang S., Cheng H., Xu L., Pei G., Wang Y., Fu C., Jiang Y., He C. (2022). Signaling pathways and targeted therapy for myocardial infarction. Signal Transduct. Target. Ther..

[97] Tardif J., Kouz S., Waters D.D., Bertrand O.F., Diaz R., Maggioni A.P., Pinto F.J., Ibrahim R., Gamra H., Kiwan G.S. (2019). Efficacy and Safety of Low-Dose Colchicine after Myocardial Infarction. N. Engl. J. Med..

[98] Ridker P.M., Everett B.M., Thuren T., MacFadyen J.G., Chang W.H., Ballantyne C., Fonseca F., Nicolau J., Koenig W., Anker S.D. (2017). CANTOS Trial Group. Antiinflammatory Therapy with Canakinumab for Atherosclerotic Disease. N. Engl. J. Med..

[99] Del Buono M., Crea F., Versaci F., Biondi-Zoccai G. (2021). NLRP3 Inflammasome: A New Promising Therapeutic Target to Treat Heart Failure. J. Cardiovasc. Pharmacol..

[100] Harada M., Van Wagoner D.R., Nattel S. (2015). Role of inflammation in atrial fibrillation pathophysiology and management. Circ. J..

[101] Gungor B., Ekmekci A., Arman A., Ozcan K.S., Ucer E., Alper A.T., Calik N., Yilmaz H., Tezel T., Coker A. (2013). Assessment of interleukin-1 gene cluster polymorphisms in lone atrial fibrillation: New insight into the role of inflammation in atrial fibrillation. Pacing Clin. Electrophysiol..

[102] Miller S.-A., Kolpakov M.A., Guo X., Du B., Nguyen Y., Wang T., Powel P., Dell’Italia L., Sabri A. (2019). A Intracardiac administration of neutrophil protease cathepsin G activates noncanonical inflammasome pathway and promotes inflammation and pathological remodeling in non-injured heart. J. Mol. Cell Cardiol..

[103] Yao C., Veleva T., Scott L., Cao S., Li L., Chen G., Jeyabal P., Pan X., Alsina K.M., Abu-Taha I. (2018). Enhanced cardiomyocyte nlrp3 inflammasome signaling promotes atrial fibrillation. Circulation.

[104] Heijman J., Muna P., Veleva T., Molina C.E., Molina C.E., Sutanto H., Tekook M., Wang Q., Abu-Taha I.H., Gorka M. (2020). Atrial Myocyte NLRP3/CaMKII Nexus Forms a Substrate for Postoperative Atrial Fibrillation. Circ. Res..

[105] Lu M., Huo Y., Tai B., Lin C., Yang H., Tsai C. (2024). Ziprasidone triggers inflammasome signaling via PI3K-Akt-mTOR pathway to promote atrial fibrillation. Biomed. Pharmacother..

[106] Yang H., Zhu J., Fu H., Shuai W. (2024). Dapansutrile Ameliorates Atrial Inflammation and Vulnerability to Atrial Fibrillation in HFpEF Rats. Heart Lung Circ..

[107] Lin A.E., Bapat A.C., Xiao L., Niroula A., Ye J., Wong W.J. (2024). Clonal Hematopoiesis of Indeterminate Potential With Loss of Tet2 Enhances Risk for Atrial Fibrillation Through Nlrp3 Inflammasome Activation. Circulation.

[108] Yang T.C., Chang P.Y., Lu S.C. (2017). L5-LDL from ST-elevation myocardial infarction patients induces IL-1beta production via LOX-1 and NLRP3 inflammasome activation in macrophages. Am. J. Physiol. Heart Circ. Physiol..

[109] Sokolova M., Sjaastad I., Louwe M.C., Alfsnes K., Aronsen J.M., Zhang L., Haugstad S.B., Bendiksen B.A., Øgaard J., Bliksøen M. (2019). NLRP3 inflammasome promotes myocardial remodeling during diet induced obesity. Front. Immunol..

[110] Suetomi T., Willeford A., Brand C.S., Cho Y., Ross R.S., Miyamoto S., Brown J.H. (2018). Inflammation and NLRP3 inflammasome activation initiated in response to pressure overload by Ca^2+^/calmodulin dependent protein kinase II delta signaling in cardiomyocytes are essential for adverse cardiac remodeling. Circulation.

[111] Cai S.-M., Yang R.-Q., Li Y., Ning Z., Zhang L., Zhou G., Luo W., Li D., Chen Y., Pan M. (2016). Angiotensin-(1–7) improves liver fibrosis by regulating the NLRP3 inflammasome via redox balance modulation. Antioxid. Redox Signal..

[112] Meng Y., Pan T., Zheng B., Chen Y., Li W. (2019). Autophagy attenuates angiotensin II induced pulmonary fibrosis by inhibiting redox imbalance-mediated NOD-like receptor family pyrin domain containing 3 inflammasome activation. Antioxid. Redox Signal..

[113] Mishra S.R., Kumar K.M., Behera B.P., Patra S., Sekhar C., Panigrahi D.P., Praharaj P.P., Singh A., Patil S., Dhiman R. (2021). Mitochondrial dysfunction as a driver of NLRP3 inflammasome activation and its modulation through mitophagy for potential therapeutics. Int. J. Biochem. Cell Biol..

[114] Qin Y.Y., Li M., Feng X., Wang J., Cao L., Shen X., Chen J., Sun M., Sheng R., Han F. (2017). Combined NADPH and the NOX inhibitor apocynin provides greater anti-inflammatory and neuroprotective effects in a mouse model of stroke. Free Radic. Biol. Med..

[115] Zhang H., Kim H., Park B.W., Noh M., Kim Y., Park J., Park J., Kim J., Sim W., Ban K. (2022). CU06-1004 enhances vascular integrity and improves cardiac remodeling by suppressing edema and inflammation in myocardial ischemia-reperfusion injury. Exp. Mol. Med..

[116] Bujak M., Kweon H.J., Chatila K., Li N., Taffet G., Frangogiannis N.G. (2008). Aging-related defects are associated with adverse cardiac remodeling in a mouse model of reperfused myocardial infarction. J. Am. Coll. Cardiol..

[117] Tarone G., Balligand J.L., Bauersachs J., Clerk A., De Windt L., Heymans S., Hilfiker-Kleiner D., Hirsch E., Iaccarino G., Knöll R. (2014). Targeting myocardial remodelling to develop novel therapies for heart failure: A position paper from the Working Group on Myocardial Function of the European Society of Cardiology. Eur. J. Heart Fail..

[118] de Boer R.A., van der Velde A.R., Mueller C., van Veldhuisen D.J., Anker S.D., Peacock W.F., Adams K.F., Maisel A. (2014). Galectin-3: A modifiable risk factor in heart failure. Cardiovasc. Drugs Ther..

[119] Lu Q., Li X., Liu J., Sun X., Rousselle T., Ren D., Tong N., Li J. (2019). AMPK is associated with the beneficial effects of antidiabetic agents on cardiovascular diseases. Biosci. Rep..

[120] Čater M., Bombek L.K. (2022). Protective Role of Mitochondrial Uncoupling Proteins against Age-Related Oxidative Stress in Type 2 Diabetes Mellitus. Antioxidants.

[121] Cadenas S. (2018). Mitochondrial uncoupling, ROS generation and cardioprotection. Biochim. Biophys. Acta Bioenerg..

[122] Bou-Teen D., Kaludercic N., Weissman D., Turan B., Maack C., Di Lisa F., Ruiz-Meana M. (2021). Mitochondrial ROS and mitochondria-targeted antioxidants in the aged heart. Free Radic. Biol. Med..

[123] Lee F., Shao P., Wallace C., Chua S., Sung P., Ko S., Chai H., Chung S., Chen K., Lu K. (2018). Combined Therapy with SS31 and Mitochondria Mitigates Myocardial Ischemia-Reperfusion Injury in Rats. Int. J. Mol. Sci..

[124] Yu H., Zhang F., Yan P., Zhang S., Lou Y., Geng Z., Li Z., Zhang Y., Xu Y., Lu Y. (2021). LARP7 Protects Against Heart Failure by Enhancing Mitochondrial Biogenesis. Circulation.

[125] Talasaz A.H., Salehiomran A., Heidary Z., Gholami K., Aryannejad H., Jalali A., Daei M. (2022). The effects of vitamin D supplementation on postoperative atrial fibrillation after coronary artery bypass grafting in patients with vitamin D deficiency. J. Card. Surg..

[126] Rodrigo R., Cereceda M., Castillo R., Asenjo R., Zamorano J., Araya J., Castillo-Koch R., Espinoza J., Larraín E. (2008). Prevention of atrial fibrillation following cardiac surgery: Basis for a novel therapeutic strategy based on non-hypoxic myocardial preconditioning. Pharmacol. Ther..

[127] Menezes-Rodrigues F.S., Errante P.R., Araújo E.A., Fernandes M.P.P., Silva M.M.D., Pires-Oliveira M., Scorza C.A., Scorza F.A., Taha M.O., Caricati-Neto A. (2021). Cardioprotection stimulated by resveratrol and grape products prevents lethal cardiac arrhythmias in an animal model of ischemia and reperfusion. Acta Cir. Bras..

[128] Hedayati N., Yaghoobi A., Salami M., Gholinezhad Y., Aghadavood F., Eshraghi R. (2023). Impact of polyphenols on heart failure and cardiac hypertrophy: Clinical effects and molecular mechanisms. Front. Cardiovasc. Med..

[129] Martins-Marques T., Rodriguez-Sinovas A., Girao H. (2021). Cellular crosstalk in cardioprotection: Where and when do reactive oxygen species play a role?. Free Radic. Biol. Med..

[130] Zinman B., Wanner C., Lachin J.M., Fitchett D., Bluhmki E., Hantel S., Mattheus M., Devins T., Johansen O.E., Woerle H.J. (2015). EMPA-REG OUTCOME Investigators. Empagliflozin, Cardiovascular Outcomes, and Mortality in Type 2 Diabetes. N. Engl. J. Med..

[131] Filippatos G., Anker S.D., Butler J., Farmakis D., Ferreira J.P., Gollop N.D., Brueckmann M., Iwata T., Pocock S., Zannad F. (2022). Effects of empagliflozin on cardiovascular and renal outcomes in heart failure with reduced ejection fraction according to age: A secondary analysis of EMPEROR-Reduced. Eur. J. Heart Fail..

[132] Saisho Y. (2020). SGLT2 Inhibitors: The Star in the Treatment of Type 2 Diabetes?. Diseases.

[133] Madonna R., Moscato S., Cufaro M.C., Pieragostino D., Mattii L., Boccio P., Ghelardoni S., Zucchi R., Caterina R. (2023). Empagliflozin inhibits excessive autophagy through the AMPK/GSK3β signalling pathway in diabetic cardiomyopathy. Cardiovasc. Res..

[134] Lee H., Shiou Y., Jhuo S., Chang C., Liu P., Jhuang W.-J. (2019). The sodium-glucose co-transporter 2 inhibitor empagliflozin attenuates cardiac fibrosis and improves ventricular hemodynamics in hypertensive heart failure rats. Cardiovasc. Diabetol..

[135] Li X., Lu Q., Qiu Y., Carmo J.M., Wang Z., da Silva A.A. (2021). Direct Cardiac Actions of the Sodium Glucose Co-Transporter 2 Inhibitor Empagliflozin Improve Myocardial Oxidative Phosphorylation and Attenuate Pressure-Overload Heart Failure. J. Am. Heart Assoc..

[136] Hsieh P., Chu M., Ching H., Huang Y., Chou W., Tsai K., Chan S. (2022). Dapagliflozin Mitigates Doxorubicin-Caused Myocardium Damage by Regulating AKT-Mediated Oxidative Stress, Cardiac Remodeling, and Inflammation. Int. J. Mol. Sci..

[137] Castelvecchio S., Frigelli M., Sturla F., Milani V., Pappalardo O.A., Citarella M., Menicanti L., Votta E. (2023). Elucidating the mechanisms underlying left ventricular function recovery in patients with ischemic heart failure undergoing surgical remodeling: A 3-dimensional ultrasound analysis. J. Thorac. Cardiovasc. Surg..

[138] Ma S., Chen L., Yan J., Shen M., Zhang R., Li M. (2022). Dapagliflozin attenuates residual cardiac remodeling after surgical ventricular reconstruction in mice with an enlarged heart after myocardial infarction. Biomed. Pharmacother..

[139] Kostin S., Krizanic F., Kelesidis T., Pagonas N. (2024). The role of NETosis in heart failure. Heart Fail. Rev..

[140] Ehrenkranz J.R., Lewis N.G., Kahn C.R., Roth J. (2005). Phlorizin: A review. Diabetes Metab. Res. Rev..

[141] Xie Y., Wei Y., Li D., Pu J., Ding H., Zhang X. (2023). Mechanisms of SGLT2 Inhibitors in Heart Failure and Their Clinical Value. J. Cardiovasc. Pharmacol..

[142] Madaan T., Akhtar M., Najmi A.K. (2016). Sodium glucose CoTransporter 2 (SGLT2) inhibitors: Current status and future perspective. Eur. J. Pharm. Sci..

[143] Bhattacharya S., Rathore A., Parwani D., Mallick C., Asati V., Agarwal S., Rajoriya V., Das R., Kashaw S.K. (2020). An exhaustive perspective on structural insights of SGLT2 inhibitors: A novel class of antidiabetic agent. Eur. J. Med. Chem..

[144] Giugliano D., Esposito K. (2019). Class effect for SGLT-2 inhibitors: A tale of 9 drugs. Cardiovasc. Diabetol..

[145] Devineni D., Morrow L., Hompesch M., Skee D., Vandebosch A., Murphy J., Ways K., Schwartz S. (2012). Canagliflozin improves glycaemic control over 28 days in subjects with type 2 diabetes not optimally controlled on insulin. Diabetes Obes. Metab..

[146] Devineni D., Curtin C.R., Polidori D., Gutierrez M.J., Murphy J., Rusch S., Rothenberg P.L. (2013). Pharmacokinetics and pharmacodynamics of canagliflozin, a sodium glucose co-transporter 2 inhibitor, in subjects with type 2 diabetes mellitus. J. Clin. Pharmacol..

[147] Yang L., Li H., Li H., Bui A., Chang M., Liu X., Kasichayanula S., Griffen S.C., Lacreta F.P., Boulton D.W. (2013). Pharmacokinetic and pharmacodynamic properties of single- and multiple-dose of dapagliflozin, a selective inhibitor of SGLT2, in healthy Chinese subjects. Clin. Ther..

[148] Tang W., Reele S., Hamer-Maansson J.E., Parikh S., de Bruin T.W. (2015). Dapagliflozin twice daily or once daily: Effect on pharmacokinetics and urinary glucose excretion in healthy subjects. Diabetes Obes. Metab..

[149] Ayoub B.M., Mowaka S., Elzanfaly E.S., Ashoush N., Elmazar M.M., Mousa S.A. (2017). Pharmacokinetic Evaluation of Empagliflozin in Healthy Egyptian Volunteers Using LC-MS/MS and Comparison with Other Ethnic Populations. Sci. Rep..

[150] Sarashina A., Koiwai K., Seman L.J., Yamamura N., Taniguchi A., Negishi T., Sesoko S., Woerle H.J., Dugi K.A. (2013). Safety, tolerability, pharmacokinetics and pharmacodynamics of single doses of empagliflozin, a sodium glucose cotransporter 2 (SGLT2) inhibitor, in healthy Japanese subjects. Drug Metab. Pharmacokinet..

[151] Sahasrabudhe V., Fediuk D.J., Matschke K., Shi H., Liang Y., Hickman A., Bass A., Terra S.G., Zhou S., Krishna R. (2019). Effect of Food on the Pharmacokinetics of Ertugliflozin and Its Fixed-Dose Combinations Ertugliflozin/Sitagliptin and Ertugliflozin/Metformin. Clin. Pharmacol. Drug Dev..

[152] Li Y., Mu Y., Shi H., Liang Y., Liu Z., Matschke K., Hickman A., Krishna R., Sahasrabudhe V. (2020). Pharmacokinetic Properties of Single and Multiple Doses of Ertugliflozin, a Selective Inhibitor of SGLT2, in Healthy Chinese Subjects. Clin. Pharmacol. Drug Dev..

[153] Kadokura T., Akiyama N., Kashiwagi A., Utsuno A., Kazuta K., Yoshida S., Nagase I., Smulders R., Kageyama S. (2014). Pharmacokinetic and pharmacodynamic study of ipragliflozin in Japanese patients with type 2 diabetes mellitus: A randomized, double-blind, placebo-controlled study. Diabetes Res. Clin. Pract..

[154] Kaku K., Isaka H., Toyoshima J., Sakatani T. (2019). Clinical pharmacology study of ipragliflozin in Japanese patients with type 1 diabetes mellitus: A phase 2, randomized, placebo-controlled trial. Diabetes Obes. Metab..

[155] Ikeda S., Takano Y., Schwab D., Portron A., Kasahara-Ito N., Saito T., Iida S. (2019). Effect of Renal Impairment on the Pharmacokinetics and Pharmacodynamics of Tofogliflozin (A SELECTIVE SGLT2 Inhibitor) in Patients with Type 2 Diabetes Mellitus. Drug Res..

[156] Rosenwasser R.F., Rosenwasser J.N., Sutton D., Choksi R., Epstein B. (2014). Tofogliflozin: A highly selective SGLT2 inhibitor for the treatment of type 2 diabetes. Drugs Today.

[157] Yaribeygi H., Atkin S.L., Butler A.E., Sahebkar A. (2019). Sodium-glucose cotransporter inhibitors and oxidative stress: An update. J. Cell Physiol..

[158] Mylonas N., Nikolaou P.E., Karakasis P., Stachteas P., Fragakis N., Andreadou I. (2024). Endothelial Protection by Sodium-Glucose Cotransporter 2 Inhibitors: A Literature Review of In Vitro and In Vivo Studies. Int. J. Mol. Sci..

[159] Sa-Nguanmoo P., Tanajak P., Kerdphoo S., Jaiwongkam T., Pratchayasa-kul W., Chattipakorn N., Chattipakorn S.C. (2017). SGLT2-inhibitor and DPP-4 inhibitor improve brain function via attenuatingmitochondrial dysfunction, insulin resistance, inflammation, andapoptosis in HFD-induced obese rats. Toxicol. Appl. Pharmacol..

[160] Pignatelli P., Baratta F., Buzzetti R., D’Amico A., Castellani V., Bartimoccia S. (2022). The Sodium-Glucose Co-Transporter-2 (SGLT_2_) Inhibitors Reduce Platelet Activation and Thrombus Formation by Lowering NOX2-Related Oxidative Stress: A Pilot Study. Antioxidants.

[161] Li C., Zhang J., Xue M., Li X., Han F., Liu X., Xu L., Lu Y., Cheng Y., Li T. (2019). SGLT2 inhibition with empagliflozin attenuates myocardial oxidative stress and fibrosis in diabetic mice heart. Cardiovasc. Diabetol..

[162] Kondo H., Akoumianakis I., Badi I., Akawi N., Kotanidis C.P., Polkinghorne M., Stadiotti I., Sommariva E., Antonopoulos A.S., Carena M.C. (2021). Effects of canagliflozin on human myocardial redox signalling: Clinical implications. Eur. Heart J..

[163] Zhang W., Lu J., Wang Y., Sun P., Gao T., Xu N. (2023). Canagliflozin Attenuates Lipotoxicity in Cardiomyocytes by Inhibiting Inflammation and Ferroptosis through Activating AMPK Pathway. Int. J. Mol. Sci..

[164] Liu I.F., Lin T., Wang S., Yen C., Li C., Kuo H., Hsieh C., Chang C., Chang C., Chen Y. (2023). Long-term administration of Western diet induced metabolic syndrome in mice and causes cardiac microvascular dysfunction, cardiomyocyte mitochondrial damage, and cardiac remodeling involving caveolae and caveolin-1 expression. Biol. Direct..

[165] Lou X., Zhang Y., Guo J., Gao L., Ding Y., Zhuo X., Lei Q., Bian J., Lei R., Gong W. (2024). What is the impact of ferroptosis on diabetic cardiomyopathy: A systematic review. Heart Fail. Rev..

[166] Hartupee J., Mann D.L. (2017). Neurohormonal activation in heart failure with reduced ejection fraction. Nat. Rev. Cardiol..

[167] Stumpf C., Seybold K., Petzi S., Wasmeier G., Raaz D., Yilmaz A., Anger T., Daniel W.G., Garlichs C.D. (2008). Interleukin-10 improves left ventricular function in rats with heart failure subsequent to myocardial infarction. Eur. J. Heart Fail..

[168] Packer M. (2018). Leptin-Aldosterone-Neprilysin Axis: Identification of Its Distinctive Role in the Pathogenesis of the Three Phenotypes of Heart Failure in People with Obesity. Circulation.

[169] De Angelis E., Pecoraro M., Rusciano M.R., Ciccarelli M., Popolo A. (2019). Cross-Talk between Neurohormonal Pathways and the Immune System in Heart Failure: A Review of the Literature. Int. J. Mol. Sci..

[170] Sugi Y., Yasukawa H., Kai H., Fukui D., Futamata N., Mawatari K. (2011). Reduction and activation of circulating dendritic cells in patients with decompensated heart failure. Int. J. Cardiol..

[171] Martini E., Kunderfranco P., Peano C., Carullo P., Cremonesi M., Schorn T., Carriero R., Termanini A., Colombo F.S., Jachetti E. (2019). Single-Cell Sequencing of Mouse Heart Immune Infiltrate in Pressure Overload-Driven Heart Failure Reveals Extent of Immune Activation. Circulation.

[172] Lee T.M., Chang N.C., Lin S.Z. (2017). Dapagliflozin, a selective SGLT2 Inhibitor, attenuated cardiac fibrosis by regulating the macrophage polarization via STAT3 signaling in infarcted rat hearts. Free Radic. Biol. Med..

[173] Elsayed M., Moustafa Y.M., Mehanna E.T., Elrayess R.A., El-Sayed N.M., Hazem R.M. (2024). Empagliflozin protects against isoprenaline-induced fibrosis in rat heart through modulation of TGF-β/SMAD pathway. Life Sci..

[174] Chen W., Zhang Y., Wang Z., Tan M., Lin J., Qian X., Li H., Jiang T. (2023). Dapagliflozin alleviates myocardial ischemia/reperfusion injury by reducing ferroptosis via MAPK signaling inhibition. Front. Pharmacol..

[175] Lu B., Nakamura T., Inouye K., Li J., Tang Y., Lundbäck P., Valdes-Ferrer S.I., Olofsson P.S., Kalb T., Roth J. (2012). Novel role of PKR in inflammasome activation and HMGB1 release. Nature.

[176] Coll R., Robertson A.A., Chae J.J., Higgins S.C., Muñoz-Planillo R., Inserra M.C. (2015). A small-molecule inhibitor of the NLRP3 inflammasome for the treatment of inflammatory diseases. Nat. Med..

[177] Lamkanfi M., Mueller J.L., Vitari A.C., Misaghi S., Fedorova A., Deshayes K., Lee W.P., Hoffman H.M., Dixit V.M. (2009). Glyburide inhibits the Cryopyrin/Nalp3 inflammasome. J. Cell Biol..

[178] He H., Jiang H., Chen Y., Ye J., Wang A., Wang C. (2018). Oridonin is a covalent NLRP3 inhibitor with strong anti-inflammasome activity. Nat. Commun..

[179] Marchetti C., Swartzwelter B., Gamboni F., Neff C.P., Richter K., Azam T., Carta S., Tengesdal I., Nemkov T., D’Alessandro A. (2018). OLT1177, a β-sulfonyl nitrile compound, safe in humans, inhibits the NLRP3 inflammasome and reverses the metabolic cost of inflammation. Proc. Natl. Acad. Sci. USA.

[180] Jiang H., He H., Chen Y., Huang W., Cheng J., Ye J. (2017). Identification of a selective and direct NLRP3 inhibitor to treat inflammatory disorders. J. Exp. Med..

[181] Huang Y., Chen Y., Wang X., Yang Y., Tao Y.J. (2018). Tranilast directly targets NLRP3 to treat inflammasome-driven diseases. EMBO Mol. Med..

[182] Cao J., Peng Q. (2022). NLRP3 Inhibitor Tranilast Attenuates Gestational Diabetes Mellitus in a Genetic Mouse Model. Drugs R D.

[183] Missmahl H.P., Riemann J. (1968). Simple determination of microangiopathy of the capillaries of the rectal mucosa in diabetics. Wien. Klin. Wochenschr..

[184] Yang L., Zhang X., Wang Q. (2022). Effects and mechanisms of SGLT2 inhibitors on the NLRP3 inflammasome, with a focus on atherosclerosis. Front. Endocrinol..

[185] Andreadou I., Daiber A., Baxter G.F., Brizzi M.F., Di Lisa F., Kaludercic N., Lazou A., Varga Z.V., Zuurbier C.J., Schulz R. (2021). Influence of cardiometabolic comorbidities on myocardial function, infarction, and cardioprotection: Role of cardiac redox signaling. Free Radic. Biol. Med..

[186] Brown A.J., Gandy S., McCrimmon R., Houston J.G., Struthers A.D., Lang C.C. (2020). A randomized controlled trial of dapagliflozin on left ventricular hypertrophy in people with type two diabetes: The DAPA-LVH trial. Eur. Heart J..

[187] Guo W., Zhao L., Huang W., Chen J., Zhong T., Yan S. (2024). Sodium-glucose cotransporter 2 inhibitors, inflammation, and heart failure: A two-sample Mendelian randomization study. J. Cardiovasc. Diabetol..

[188] Stachteas P., Nasoufidou A., Karagiannidis E., Patoulias D., Karakasis P., Alexiou S., Samaras A., Zormpas G., Stavropoulos G., Tsalikakis D. (2024). The Role of Sodium Glucose Co-Transporter 2 Inhibitors in Atrial Fibrillation: A Comprehensive Review. J. Clin. Med..

[189] Zarei B., Fazli B., Tayyebi M., Teshnizi M.A., Moeinipour A., Javedanfar O., Bayaz R.J.D., Rahmati M., Ghavami V., Amini S. (2024). Evaluation of the effect of empagliflozin on prevention of atrial fibrillation after coronary artery bypass grafting: A double-blind, randomized, placebo-controlled trial. Naunyn-Schmiedeberg’s Arch. Pharmacol..

[190] Aghakouchakzadeh M., Hosseini K., Haghjoo M., Mirzabeigi P., Tajdini M., Talasaz A.H., Jalali A., Askarinejad A., Kohansal E., Hedayat B. (2024). Empagliflozin to prevent post-operative atrial fibrillation in patients undergoing coronary artery bypass graft surgery: Rationale and design of the EMPOAF trial. Pacing Clin. Electrophysiol..

[191] Yaribeygi H., Butler A.E., Atkin S.L., Katsiki N., Sahebkar A. (2018). Sodium-glucose cotransporter 2 inhibitors and inflammation in chronic kidney disease: Possible molecular pathways. J. Cell Physiol..

[192] Mozaffarian D., Marchioli R., Macchia A., Silletta M.G., Ferrazzi P., Gardner T.J., Latini R., Libby P., Lombardi F., O’Gara P.T. (2012). Fish oil and postoperative atrial fibrillation: The Omega-3 Fatty Acids for Prevention of Post-operative Atrial Fibrillation (OPERA) randomized trial. JAMA.

[193] Corradi D., Saffitz J., Novelli D., Asimaki A., Simon C., Oldoni E., Masson S., Meessen J.M., Monaco R., Manuguerra R. (2020). Prospective Evaluation of Clinico-Pathological Predictors of Postoperative Atrial Fibrillation: An Ancillary Study From the OPERA Trial. Circ. Arrhythm. Electrophysiol..

[194] Neal B., Perkovic V., Mahaffey K.W., De Zeeuw D., Fulcher G., Erondu N., Wayne Shaw D.S.L., Law G., Desai M., Matthews D.R. (2017). Canagliflozin and cardiovascular and renal events in type 2 diabetes. N. Engl. J. Med..

[195] Uthman L., Baartscheer A., Schumacher C.A., Fiolet J.W., Kuschma M.C., Hollmann M.W., Coronel R., Weber N.C., Zuurbier C.J. (2018). Direct Cardiac Actions of Sodium Glucose Cotransporter 2 Inhibitors Target Pathogenic Mechanisms Underlying Heart Failure in Diabetic Patients. Front. Physiol..

[196] Chilton R.J. (2020). Effects of sodium-glucose cotransporter-2 inhibitors on the cardiovascular and renal complications of type 2 diabetes Diabetes. Obes. Metab..

[197] Palmiero G., Cesaro A., Vetrano E., Pafundi P.C., Galiero R., Caturano A., Moscarella E., Gragnano F., Salvatore T., Rinaldi L. (2021). Impact of SGLT2 Inhibitors on Heart Failure: From Pathophysiology to Clinical Effects. Int. J. Mol. Sci..

[198] Kang S., Verma S., Hassanabad A.F., Teng G., Belke D.D., Dundas J.A., Guzzardi D.G., Svystonyuk D.A., Pattar S.S., Park D.S. (2020). Direct Effects of Empagliflozin on Extracellular Matrix Remodelling in Human Cardiac Myofibroblasts: Novel Translational Clues to Explain EMPA-REG OUTCOME Results. Can. J. Cardiol..

[199] Byrne N.J., Matsumura N., Maayah Z.H., Ferdaoussi M., Takahara S., Darwesh A.M., Levasseur J.L., Jahng J.W., Vos D., Parajuli N. (2020). Empagliflozin Blunts Worsening Cardiac Dysfunction Associated with Reduced NLRP3 (Nucleotide-Binding Domain-Like Receptor Protein 3) Inflammasome Activation in Heart Failure. Circ. Heart Fail..

[200] Inzucchi S.E., Zinman B., Fitchett D., Wanner C., Ferrannini E., Schumacher M., Schmoor C., Ohneberg K., Johansen O.E., George J.T. (2018). How Does Empagliflozin Reduce Cardiovascular Mortality? Insights From a Mediation Analysis of the EMPA-REG OUTCOME Trial. Diabetes Care..

[201] Ceriello A., Ofstad A., Zwiener I., Kaspers S., George J., Nicolucci A. (2020). Empagliflozin reduced long-term HbA1c variability and cardiovascular death: Insights from the EMPA-REG OUTCOME trial. Cardiovasc. Diabetol..

[202] Anker S., Butler J., Filippatos G.S., Jamal W., Salsali A., Schnee J., Kimura K., Zeller C., George J., Brueckmann M. (2019). Evaluation of the effects of sodium-glucose co-transporter 2 inhibition with empagliflozin on morbidity and mortality in patients with chronic heart failure and a preserved ejection fraction: Rationale for and design of the EMPEROR-Preserved Trial. Eur. J. Heart Fail..

[203] Hundertmark M., Agbaje O.F., Coleman R. (2021). Design and rationale of the EMPA-VISION trial: Investigating the metabolic effects of empagliflozin in patients with heart failure. ESC Heart Fail..

[204] Kim S.R., Lee S.G., Kim S.H., Kim J.H., Choi E., Cho W., Rim J.H., Hwang I., Lee C.J., Lee M. (2020). SGLT2 inhibition modulates NLRP3 inflammasome activity via ketones and insulin in diabetes with cardiovascular disease. Nat. Commun..

